# Weight change, cardio-metabolic risk factors and cardiovascular incidence in people with serious mental illness: protocol of a population-based cohort study in the UK from 1998 to 2020

**DOI:** 10.1136/bmjopen-2021-053427

**Published:** 2021-11-03

**Authors:** Charlotte Lee, Felicity Waite, Margaret C Smith, Min Gao, Clare Bankhead, Paul Aveyard, Carmen Piernas

**Affiliations:** 1 Nuffield Department of Primary Care Health Sciences, University of Oxford, Oxford, UK; 2 NIHR Oxford Biomedical Research Centre, Oxford University Hospitals NHS Foundation Trust, Oxford, UK; 3 Department of Psychiatry, University of Oxford, Oxford, UK; 4 Oxford Health NHS Foundation Trust, Oxford, UK; 5 School of Public Health, Peking University Health Science Centre, Beijing, China

**Keywords:** schizophrenia & psychotic disorders, primary care, epidemiology

## Abstract

**Introduction:**

People with serious mental illness (SMI), which includes people with diagnoses of schizophrenia spectrum and bipolar disorders, face significant health inequality. This includes a life expectancy reduced by 15–20 years mostly due to premature cardiovascular disease (CVD) compared with the general population. Excess weight gain and related comorbidities are preventable risk factors for CVD. To improve the understanding and management of CVD in people with SMI, we will examine the association between SMI and: (1) weight change; (2) cardio-metabolic risk factors for CVD; and (3) incidence of and mortality from CVD. We will also (4) examine the incidence of referral to weight management services for people with SMI compared with people without SMI.

**Methods and analysis:**

In this retrospective cohort study, we will link general practice records from the UK Clinical Practice Research Datalink Aurum database. We will establish a cohort of patients diagnosed with SMI between 1998 and 2020 who are matched with up to four controls on age, sex, general practice and calendar year. We will use multivariable mixed-effects linear regression models and Cox proportional hazard models with sequential adjustment for potential confounders identified by separate directed acyclic graphs.

**Ethics and dissemination:**

This study has been reviewed and approved by the Independent Scientific Advisory Committee for Medicines and Healthcare products Regulatory Agency database research. The results will be published in a peer-reviewed journal.

**Strengths and limitations of this study**

- The key strengths of the study include the representative population contained in the Clinical Practice Research Datalink Aurum, the sizeable and linked dataset, and substantial statistical power.
- However, limitations include its observational nature and potential for residual confounding due to lack of information on some factors.
- There is also potential for selection bias since physical health checks are not always performed on random population samples.

## Introduction

Serious mental illness (SMI) includes psychotic disorders like schizophrenia spectrum and bipolar disorders, which are marked by hearing, seeing, or believing things that are not real.^1^ People with SMI have a life expectancy that is approximately 15–20 years lower than the general population.^2^ Two-thirds of premature mortality in this group is explained by preventable cardiovascular disease (CVD).^3^ A 2017 meta-analysis reported people with SMI have a significantly increased incidence of and mortality from than the general population: HR: 1.78, 95% CI 1.60, 1.98.^4^ This indicates an important clinical need to identify the factors underlying the higher incidence of CVD for this group and routes for prevention and treatment.^5 6^

Overweight (body mass index (BMI) 25–29.9 kg/m^2^) and obesity (BMI≥30 kg/m^2^) are causally linked to cardio-metabolic abnormalities like insulin resistance, dyslipidaemia and hypertension, which are major preventable risk factors for CVD. Latest figures show that 64% of adults in the UK are classified as overweight or obese.^7^ Overweight and obesity have not been consistently reported for people with SMI. A 2018 report based on a representative sample of English general practice records found that people with SMI were three times more likely to have obesity between the ages 15–34, and 1.6 times more likely for ages 55–74 compared with the general population.^8^ However, these estimates were limited by high rates on non-completion of BMI in general practice. Moreover, other estimates are often drawn from cross-sectional analyses and often include small numbers of people attending specific services within a geographical area with no comparison group.^4^

The factors underlying excess weight gain in people with SMI are multifactorial. Psychotropic drugs (ie, antipsychotics, antidepressants, mood stabilisers) are the mainstay treatment for the symptoms of psychosis and affect weight.^9^ Evidence from clinical trials has linked these drugs to rapid weight gain, but the long-term effects after a diagnosis are unclear.^9^ In one 11-year follow-up study of people with schizophrenia, increased CVD mortality was reported in people treated with quetiapine (adjusted HR: 1.41, 95% CI 1.09 to 1.82) and reduced CVD mortality in people treated with clozapine (adjusted HR: 0.74, 95% CI 0.60 to 0.91).^10^ However, these estimates may result from unmeasured confounding by socioeconomic and other demographic factors.^11^ Accordingly, a better understanding of the long-term impact of medication on weight change, cardio-metabolic risk factors, and incidence of and mortality from CVD is warranted.

Other factors that cause weight gain include unhelpful dietary patterns and physical inactivity. These behaviours are more common in people with SMI compared with the general population and may reflect the social disadvantage to which SMI contributes.^12^ In addition, the prevalence of smoking is estimated to be twice that in people with SMI compared with general population,^13^ and evidence shows people with schizophrenia smoke more cigarettes per day and more intensely than the general population of smokers.^14^ Other studies using polygenic risk scores for liability to schizophrenia have found no association between those scores and physical health outcomes except for conditions mediated through obesity and smoking.^15^ This suggests that the excess risk to people with SMI are modifiable.

Since obesity is modifiable, US and UK clinical guidelines recommend intervening to prevent and manage obesity. In the UK, the first-line intervention is general practitioner (GP) advice or a referral to a weight management programme to support an energy-restricted diet and increase physical activity for people with a BMI >25 kg/m^2^.^16 17^ In the general population, trials of such programmes lead to greater weight-loss than self-directed attempts.^18^ Another study in the general population reported the rates of referral between 2005 and 2012 was 20.0 (95% CI 19.3 to 20.8) per 1000 person-years, compared with weight-related advice at 30.3 (29.3 to 31.4).^19^ This rate is low, and although these interventions may not be recorded, other evidence also point to low rates of intervention.^20^ However, we have no data on whether GPs are using the annual review of people with SMI, which focuses specifically on preventative healthcare, to provide support for weight management such as referrals to weight management programme.

We have been unable to find studies that have followed people with SMI over several years to examine how weight and cardio-metabolic profiles change. It is possible these relate to ongoing psychotropic and/or weight management programmes. The longest study to date was an 18-month trial of people with schizophrenia. In this study, participants randomised to olanzapine had greater increases in weight, glycaemia and adverse lipid profile than other antipsychotics.^21^ Randomised controlled trials (RCTs) are often limited to short follow-up of selected populations, and there are methodological issues surrounding imputation strategies used to address high dropout rates.^22 23^ Thus, it remains unclear how much weight and cardio-metabolic profiles change over the longer term, and how much of the excess incidence and mortality from CVD is explained by those changes in people with SMI.

In this protocol, we outline our plans to address this gap in a large, retrospective, population-based study in people with SMI matched with controls without SMI in the UK from 1998 to 2020. Our objectives are to:

- Examine the association between SMI and weight change.
- Examine the association between SMI and change in cardio-metabolic risk factors for CVD.
- Examine the association between SMI and incidence of and mortality from CVD.
- Compare incidence of advice and offer and actual referral to weight management services in people with both SMIs and BMI ≥25 kg/m^2^ with controls.

## Methods and analysis

### Patient and public involvement

We consulted 12 members of the public with lived experience of SMI to ensure the outcomes that matter to them are included in the protocol. Their continued involvement will inform the dissemination of our results.

### Design

This protocol is written according to the ‘REporting of studies Conducted using Observational Routinely-collected health Data’ statement.^24^ We will use a retrospective cohort using individual patient data from a large primary care database to examine all objectives. The planned study start date is 01 September 2021 and end date is 31 August 2024.

### Data source

We will use the Clinical Practice Research Datalink (CPRD) Aurum—a governmental, not-for-profit research service holding anonymised primary care records drawn from participating general (primary care) practices dating back to 1987 (http://www.cprd.com). To date, it encompasses routinely collected patient data records of around 16 million patients who are currently registered with some 700 participating general practices. Patient demographic data (ie, year of birth, sex and ethnicity), characteristics (ie, height, weight, smoking status), symptoms, clinical diagnoses, consultations, referrals, prescribed medications and results of investigations are available.

### Data linkage

Data will be linked to the Hospital Episode Statistics (HES) and the Office for National Statistics mortality databases. Data linkage to the practice level Index of Multiple Deprivation (IMD), as a proxy measure for socioeconomic status (SES), will also be used when describing the baseline characteristics in the descriptive analyses and in secondary analyses.

### Population

#### Source population

The source population will be selected from the CPRD Aurum. The source population will include all UK National Health Service (NHS) patients (male/female) registered with a participating general practice who are eligible for data linkage. We will not include up-to-standard records because this is not yet available as a measure of data quality in the CPRD Aurum database.

#### Study cohort

We will select a study cohort from the CPRD Aurum to investigate objectives 1–3. We will identify and extract the records in the primary care and HES dataset for all patients with a diagnosis of SMI (hereafter *exposed*) and who are eligible for data linkage. Diagnostic Read codes, which are the standard clinical codes used in UK general practice, will be used to identify an SMI diagnosis. We will also use codes according to the International Classification of Diseases version 10 (ICD-10) from the HES data. The date of the first SMI Read code will be used as the index date, which is the earliest date that an exposed patient may enter the study. From the study cohort, a subcohort of patients will be selected to investigate objective 4. To enter the subcohort, the exposed patient needs a recorded BMI ≥25 kg/m^2^ at any time after the index date. All information will be extracted for the period 01 January 1998 to 31 October 2020—the earliest and latest dates respectively that data are available.

#### Exclusion criteria

Patients will be excluded from the cohort(s) if they: have less than 1 year of active data in their participating practice before the index date; and/or who have a Read code for coronary heart disease, congestive heart failure or cardiomyopathy, and/or stroke before the index date. A flow diagram will present the selection and exclusion (with reasons where possible) of patients in the study.

#### Duration of follow-up

Patients will be followed up in the database from their index date until the earliest of the following: date of death; date of outcome of interest; date of leaving the participating practice; date of the latest download of data; or the end of the study follow-up period (ie, up to 22 years).

#### Comparison group

The unexposed represents the counterfactual and will include patients without an SMI Read code ever (hereafter *unexposed*) drawn from the source population. Up to four unexposed matches will be selected at baseline only if they have a qualifying weight Read code within±one year of the index date to the exposed in which they are matched. The matching variables include: age, sex, practice and calendar time. All weight Read codes will be extracted.

### Exposures

For all analyses, the exposure of interest is SMI. We will extract information on the study cohort up to censoring. Extracted information includes the diagnosis date and type, which will be grouped according to the two major SMI subgroups as classified by the ICD-10: F20-29 non-affective psychotic disorder (ie, schizophrenia, schizotypal and delusional disorders, psychosis not otherwise specified) and F30-39 affective psychotic disorder (ie, bipolar, manic, schizoaffective disorders, major depressive disorder with psychosis). Patients may have Read codes in the primary care data relating to both non-affective and affective psychotic disorders. These will be classified using the most recent Read code because we will assume that the latest diagnosis is the correct one. For example, it is common for diagnoses to be clarified over time from first episode psychosis to schizophrenia. Based on similar studies, instances where there are Read codes relating to both diagnoses at the same date will be coded under non-affective psychotic disorder.^25^ Data on individual prescriptions for psychotropic medication will be extracted from the primary care data from index date until censored.

### Outcomes

The primary aim of objective 1 is to assess the impact of an SMI diagnosis on weight change. A pilot search of the database indicated waist circumference was not reliably recorded so we did not include this as a measure of adiposity. The outcome for objective 2 is change in cardio-metabolic risk factors for CVD, which include change in systolic and diastolic blood pressure, change in total cholesterol/high-density lipoprotein ratio, and change in glycaemia indicated by HbA1c or fasting glucose. The outcome for objective 3 is incidence of and mortality from CVD. The objective for 4 is coded weight management advice and referral to weight management services. We will interpret the data based on the associations for all outcomes, as well as consistency and interpretable patterns. The specific outcomes for each objective are outlined in table 1.

**Table 1:** Outcome measures

Objective	Outcome	Measures
Objective 1: Examine the association between SMI and weight change	Weight change	Absolute mean weight change (kg)
		Percentage weight change (kg)
		Change in body mass index (kg/m^2^)
Objective 2: Examine the association between SMI and changes cardio-metabolic risk factors for CVD	Cardio-metabolic risk	Change in fasting blood glucose (FBG)
		Change in haemoglobin A1c (HbA1c)
		Change in mean total-cholesterol
		Change in triglycerides
		Change in low-density lipoprotein (LDL)
		Change in blood pressure (systolic (SBP) and diastolic (DBP))
Objective 3: Total incident and fatal CVD	Incidence of CVD and CVD fatalities	Angina diagnosed by GP
		Acute coronary syndrome
		Coronary heart disease
		Congestive heart failure
		Cardiomyopathy
		Total stroke
		Total fatal CVD events
Objective 4: Weight management advice and service referral	Proportion (%) of exposed and unexposed who are recorded as offered advice, and then offered and actually referred to a weight management service	

CVD, cardiovascular disease; GP, general practitioner; SMI, serious mental illness.

### Confounding

We have identified confounding variables using directed acyclic graphs (DAGs), which aims to theoretically formalise potential confounders, colliders and biasing relationships.^26^ The DAG for each objective is described in online supplemental material S1. The DAG for objective 1 shows one direct path from the exposure (ie, SMI) to the outcome (ie, weight change), and four indirect paths: via health behaviours (ie, alcohol use, smoking and diet), SES status, psychotropic medication, and healthcare access and provision. Diet and physical activity are latent variables in CPRD Aurum because they are unmeasured. There are three confounding paths: via age, sex and ethnicity.^27 28^ Thus, the minimally sufficient adjustment for the total effect of SMI status on weight is to adjust for age, sex, and ethnicity.

10.1136/bmjopen-2021-053427.supp1 Supplementary data

Frequency matching will ensure our ability to adjust for confounding variables that include age, sex, and calendar year, and matching on practice will remove practice-specific variables. In addition, we will fully adjust our models by considering several covariates in the relationship between the exposure and outcome (see table 2). All categorical variables will be analysed using the natural reference category as the reference group. Data on individual prescriptions for psychotropic and cardio-metabolic medications will be extracted from the primary care data from index date until censoring. For psychotropic medications, drugs will be classified according to the British National Formulary. For objectives 2 and 3, we will assess the impact of weight gain on change in cardio-metabolic risk factors and incidence of CVD using mediation analysis to allow us to estimate the direct (unmediated) and indirect (mediated) effects of SMI on CVD risk and incidence. To do this, we will fit two regression models: one regressing the outcome on the exposure, adjusting for confounders and another regressing the mediator on the exposure and confounders using the Stata *Med4way* command to estimate mediated effects.^29^ For objective 4, we will assess the presence of confounding by comparing estimates with and without adjustment. We will use Read codes, which have been previously developed by our team, to identify outcome and covariate records.

**Table 2:** Confounders

Confounders and covariates		Measures
Health behaviours	Smoking status	Categorical (0=never; 1=ex-smoker; 2=current smoker (light: 1–9 cigarettes/day; moderate: 10–19 cigarettes/day; heavy: 20+ cigarettes/day)). This is coded in GP records. We will use the most recent recorded value of smoking status at the study start date.
	Alcohol use	Continuous (units per week). This is coded in GP records. We will use the most recent recorded value of alcohol use at the study start date.
Demographics	Socioeconomic status (SES)*	Categorical (quintiles)
	Ethnicity†	Categorical (white; Asian or Asian British; black or black British; other ethnic group, mixed)
	Sex†	Binary (0=male; 1=female)
	Age at study entry, in years	Continuous
Physical and/or medical factors	Baseline weight status (body mass index (BMI))	Categorical (normal weight (BMI 18.5–24.9 kg/m^2^); overweight (25.0–29.9); obese (30.0–34.9); severe obesity (35.0–35.9); morbid obesity (40.0–44.9)
	Psychotropic medication	Categorical (antipsychotics (first-generation and second-generation); antidepressants; mood stabilisers)
	Cardio-metabolic medication	Categorical (blood glucose lowering medications; antihypertensive treatments, statins)
We include the following additional variables for objectives 3 and 4 only		
CVD risk factors	Total cholesterol/HDL ratio	Continuous
	Systolic and diastolic blood pressure	Continuous
	Glycaemia	HbA1c/fasting blood glucose (FBG)

*This variable is grouped according to practice level Index of Multiple Deprivation. This is an indicator of material deprivation based on income; employment; health deprivation and disability; education, skills and training; crime; barriers to housing and services; and living environment. It is categorised as quintiles 1–5 with 1 being the most deprived and 5 being the least deprived.

†This variable is grouped according to recommendations by the UK Office for National Statistics.^33 34^

CVD, cardiovascular disease; GP, general practitioner; HbA1c, haemoglobin A1c; HDL, high-density lipoprotein.

### Variable library

Exposures, outcomes and covariates of interest will be identified using medical coding (Read/SNOMED/EMIS), product coding (DM+D) and recording of patient death or transfer out of a participating practice in CPRD Aurum. The final definitions and code library has been drawn up and validated through consensus agreement among all investigators.

### Sample size calculation

The estimated median follow-up time for the *exposed* with a weight Read code in their record in the 1 year *before* the index date is 3.2 years (mean: 4.6), which is based on our feasibility work for this project. We assumed a 1% annual incidence rate of CVD meaning 3% of the study population would develop CVD over the 3.2 years of follow-up.^30^ We also assumed a relative risk (RR) of 1.3 for CVD in the *exposed* versus *unexposed*.^31^ The n ratio was set at 0.25, which achieved the best balance between statistical power and data cost. Our calculation suggested a minimum sample size of n=3907 at 80% power to detect an RR of 1.3, alpha <0.05%. Our feasibility estimates indicate that there are 135 870 patients with SMI in CPRD Aurum, meaning the study is sufficiently powered to detect small effects.

### Statistical analysis

Analyses will be conducted in Stata using an estimation approach. We do not intend to impute missing data. The entry date into the analysis is the index date. The outcome date is the earliest of: (1) the date of death or (2) date of diagnosis of the outcome of interest. For objectives 1 and 2, we will use all repeated measures and run separate models for each analyses. For objective 3, we will use the first recorded diagnosis of the outcome of interest rather than recurrent events. Patients who do not have the outcome of interest will be censored at the earliest of: (1) date of death, (2) date of leaving the practice, (3) date of the latest download of data or (4) the study end date.

#### Descriptive summaries

Frequency matching will reduce potential confounding and balance the baseline characteristics of the *exposed* and *unexposed* on age, sex, practice and calendar year. Adequacy of matching will be confirmed by comparing means using independent t-tests. Descriptive statistics will describe the baseline characteristics of patients in the cohort. Categorical data will be presented as frequency and percentages, and continuous variables will be summarised using descriptive statistics mean, standard deviation (SD) or median and interquartile range (IQR). Comparisons between the groups will be made using χ^2^ tests and t-tests (or Wilcoxon rank-sum tests) as appropriate. Length of exposure and the most commonly prescribed class of psychotropic for each patient will be summarised. The causes of death will be categorised as CVD and all-cause mortality.

#### Primary analyses

For objectives 1 and 2, the outcome will be visually inspected by plotting trajectories of weight and cardio-metabolic risk factor change using all repeated measurements for patients with SMI versus patients without SMI. For these analyses, we will use a mixed-effects linear regression model including terms for SMI, time and their interaction to estimate regression coefficients with 95% CIs by SMI status. Models incorporating psychotropic medications will analogously include medication-time multiplicative interaction terms. We will include a random effect for individual and practice identifier to account for intra-class correlation resulting from common practice membership. Diagnostic tests will be conducted to confirm linear regression assumptions are met including linearity of residuals, residual normality using quantile–quantile (Q–Q) plots, and residual heteroscedasticity using residual versus fitted value plots. We will assess for exploratory subgroup effects by adding interactions (eg, by age, sex, ethnicity and SES) and, if significant, we will assess the strength of the associations in separate subgroups for clarity. For objective 2, we will model the association between weight and cardio-metabolic change using restricted cubic splines to allow for non-linear associations. For objective 3, we will use Kaplan-Meier survival estimates for the exposed and unexposed groups to estimate the distribution of time from index date to total and fatal CVD and mortality (all-cause). The statistical equivalence of the two curves will be examined using the log-rank test. This analysis will use a survival from index date between the exposed and unexposed groups using an adjusted Cox proportional hazards regression model with follow-up time as the timescale. Restricted cubic splines will be computed with five knots to visually explore non-linear associations between the exposure and the outcomes. For objective 4, we will use a Poisson regression examining person-time to estimate the rate at which patients who received weight management services over the study period in patients with SMI compared with patients who do not have SMI, adjusting for confounders and BMI.

#### Sensitivity and secondary analyses

If necessary, data on missing confounders and covariates will be addressed by adjusting for missingness as a category. Sensitivity analyses will examine the impact of running the analyses with everyone included as discussed above and confined only to those with complete data. A second sensitivity analysis will be performed excluding patients that experience the outcome within 2 years of the SMI Read code to avoid reversed causality from other underlying disease.

## Ethics and dissemination

This study has been approved by the Independent Scientific Advisory Committee (ISAC) to the Medicines and Healthcare Products Regulatory Agency (reference: 20_000186). To guarantee the confidentiality of patient information, only the authors will have access to the data during the study. The full statistical code will be available from the authors after the publication of the results. The results of this study will be presented at scientific conferences and published in peer-reviewed journals.

## Discussion

In the UK, the NHS Long Term Plan commits the health service to greater action on reducing inequalities for the most overlooked groups. This study responds to this national agenda by focusing on the major cause of death for people with SMI—cardiovascular disease. We plan to leverage the power of a large UK primary care database to provide new insight on how these actions may be achieved.

Our results will estimate weight change, its risk factors including psychotropic medication, and its management in primary care as a preventative target for CVD. This could inform medication choice for people with SMI and clinicians, particularly if there are meaningful differences between drugs. In addition, our results will estimate the change in cardio-metabolic factors in people with SMI relative to those without SMI. This will be valuable to policy-makers because these are key risk factors for CVD that are being addressed through the NHS Long Term Plan. Furthermore, identifying the factors underlying the higher incidence of CVD will help us understand the degree to which these factors are valuable therapeutic targets. Lastly, understanding whether people with SMI are being offered support to manage their weight will inform national initiatives to overcome therapeutic nihilism. It is also important to understand whether there are differences in response between people with SMI and the general population, which would inform us about whether interventions need to be tailored to people with SMI for more personalised care. Ultimately, the results will offer a foundation to assess the degree to which the current focus on CVD prevention in people with SMI is having clinical benefits.

Since this study is observational, there are inherent strengths and limitations that are discussed. A strength of the study is its use of a representative, validated and sizeable dataset. Furthermore, the CPRD Aurum is an appropriate data source using primary care records. This will minimise selection, recall and respondent bias since UK general practices tend to have good levels of accuracy in recording prescriptions and clinical diagnoses. The results will also have direct and important applications to international guidelines on patient healthcare management, as well as to British primary care physicians, mental health services and members of the public with experience of SMI to initiate individual action. Finally, the longitudinal design is useful in addressing important clinical questions not usually possible within RCTs, and a matched-cohort improves efficiency since we will not need to include all controls, while tackling confounding.

Since the study is descriptive, any association observed would not imply an SMI diagnosis is causative of adverse physical health outcomes. However, we might assume the greater the magnitude of the observed association the less likely it is due to bias and more likely due to a causal process.^32^ Furthermore, diet and physical activity are unmeasured confounders because they are unobserved in CPRD Aurum. Similarly, other unmeasured confounders like poverty may place people with SMI at increased risk of homelessness, which has consequences on healthcare access and provision. We address this potential confounding by linking to patient level IMD as a proxy measure of SES, but acknowledge this may not fully capture deprivation and leaves room for residual confounding. Moreover, information bias may be present since the CPRD Aurum relies on a GP recording of health outcomes. Furthermore, we can only examine primary care prescriptions. Any prescribing solely within secondary care may not be included as this was not historically routinely recorded in GP records. Thus, it is possible we will miss the first prescription(s). Finally, physical health checks are not always performed on random samples of the population, and it is possible that more regular testing is done on people with overweight and obesity. We expect that some patients will have incomplete information recorded. However, by determining what proportion of patients do not have appropriate information recorded, we can evaluate the scale of these problems, and highlight the need for improved recording for continuity of care between healthcare professionals.

## Supplementary Material

Author's manuscript
