# Supplementary material for: Weight change, cardio-metabolic risk factors and cardiovascular incidence in people with serious mental illness: protocol of a population-based cohort study in the UK from 1998 to 2020
Source: BMJ Open. 2021 Nov 3;11(11):e053427. doi: 10.1136/bmjopen-2021-053427 (PMC8572405; doi:10.1136/bmjopen-2021-053427)
Supplement: Supplementary data [file bmjopen-2021-053427supp001.pdf]

Weight change, cardio-metabolic risk factors, and cardiovascular incidence in people with serious mental illness: protocol of a population-based cohort study in the UK from 1997 – 2020

Supplementary Material

Supplementary material S1. Direct acyclic graphs (DAGs) for the association between SMI and each outcome.

In the following figures, the green box (i.e., SMI status) is the exposure variable. The blue box 'I' is the outcome variable. The grey boxes 'diet' and 'physical activity' are latent variables because they are unobserved in the CPRD Aurum dataset. Causal paths are indicated via green arrows; biasing paths in red; and paths with no influence on the outcome in black.

Figure 1. DAG for the association between SMI and mean weight change (objective 1).

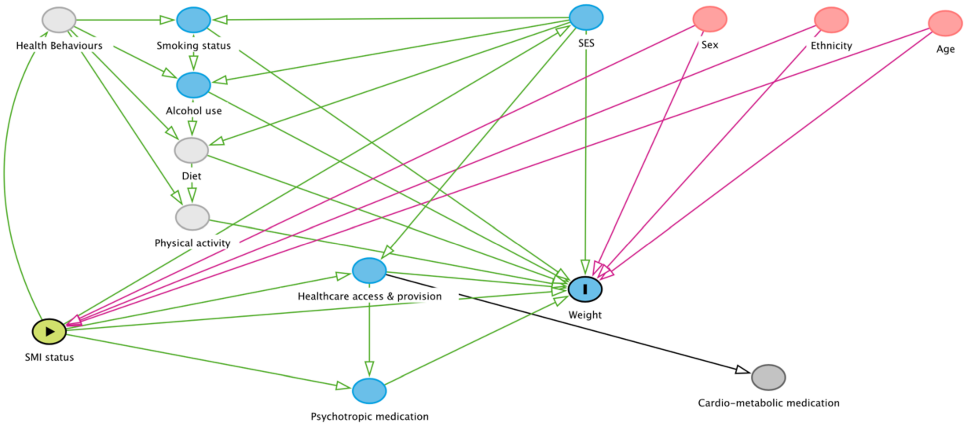

This DAG shows **five** paths between the exposure (i.e., SMI status) and outcome (i.e., weight): the (1) direct path from SMI status to weight; the indirect paths via (2) health behaviours (i.e., smoking status, alcohol use, diet, and physical activity), via (3) socio-economic status (SES), via (4) psychotropic medications, and via (5) healthcare access and provision. All variables on the indirect paths are mediating. There are three confounding paths: the path via age, sex, and ethnicity. Thus, the minimally sufficient adjustment to get the total effect of the exposure on the outcome is to adjust for age, sex, and ethnicity. The direct effect cannot be estimated by covariate adjustment.

Figure 2. DAG for the association between SMI and biomarkers for CVD risk (objective 2).

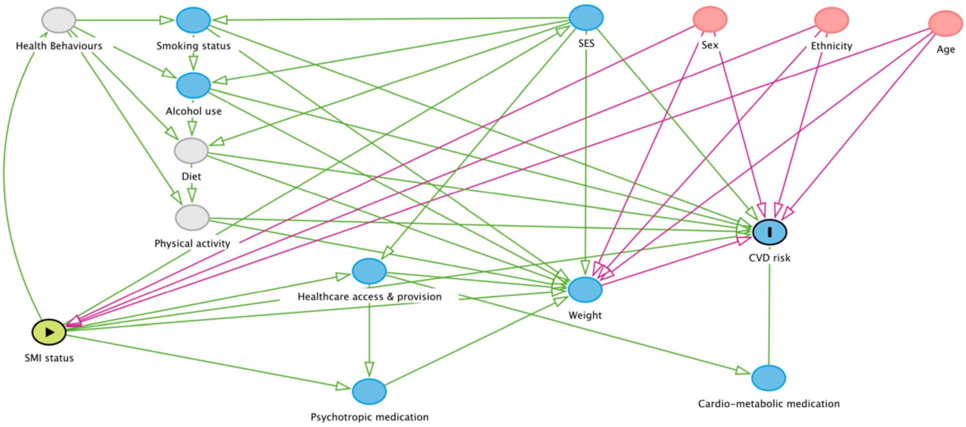

This DAG shows **seven** paths between the exposure (i.e., SMI status) and outcome (i.e., CVD risk): the (1) direct path from SMI status to CVD risk; the indirect paths via (2) health behaviours (i.e., smoking status, alcohol use, diet, physical activity), via (3) SES, via (4) psychotropic medications, via (5) healthcare access and provision, via (6) cardio-metabolic medication, and via (7) weight. There are three confounding paths: the path via age, sex, and ethnicity. Thus, the minimally sufficient adjustment to get the total effect of the exposure on the outcome is to adjust for age, sex, and ethnicity. The direct effect cannot be estimated by covariate adjustment.

Figure 3. DAG for the association between SMI and CVD incidence (objective 3).

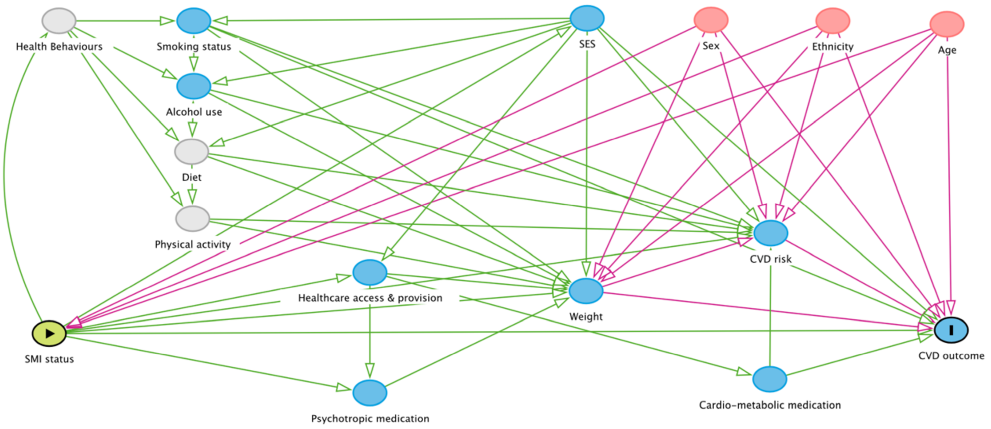

This DAG shows **eight** paths between the exposure (i.e., SMI status) and outcome (i.e., CVD risk): the (1) direct path from SMI status to CVD risk; the indirect paths via (2) health behaviours (i.e., smoking status, alcohol use, diet, and physical activity), via (3) SES, via (4) psychotropic medications, via (5) healthcare access and provision, via (6) cardio-metabolic medication, via (7) weight, and via (8) CVD risk. The minimally sufficient adjustment to get the total effect of the exposure on the outcome is to adjust for age, sex, and ethnicity. The minimally sufficient adjustment for estimating the direct effect of the exposure on the outcome is to adjust for: age, sex, ethnicity, SES, smoking, weight, cardio-metabolic medication, and CVD risk.

Figure 4. DAG to compare the prevalence of offer and actual referral to weight management services in people with both SMI and obesity with controls (objective 4).

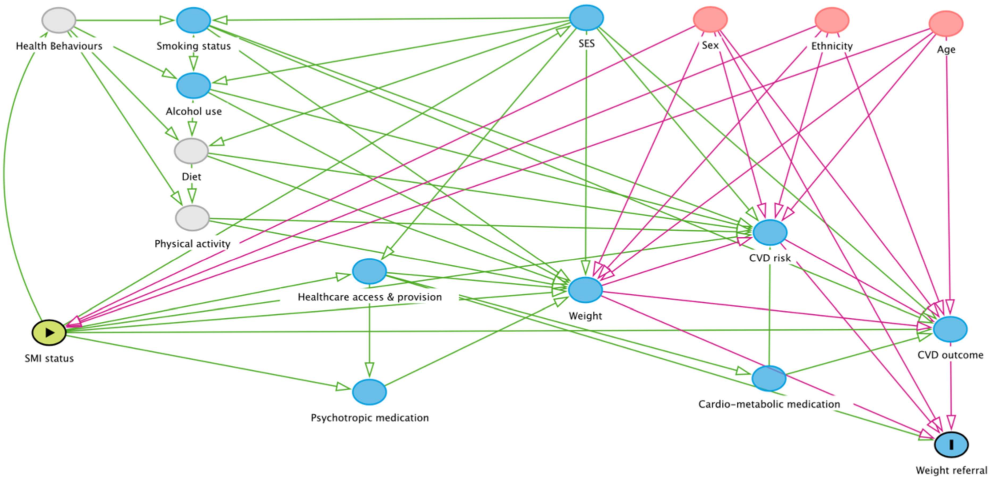

This model does not change the adjustment set.
