# Supplementary material for: Weight change, cardio-metabolic risk factors and cardiovascular incidence in people with serious mental illness: protocol of a population-based cohort study in the UK from 1998 to 2020
Source: BMJ Open. 2021 Nov 3;11(11):e053427. doi: 10.1136/bmjopen-2021-053427 (PMC8572405; doi:10.1136/bmjopen-2021-053427)
Supplement: Author's manuscript [file bmjopen-2021-053427.draft_revisions.pdf]

BMJ Open is committed to open peer review. As part of this commitment we make the peer review history of every article we publish publicly available.

When an article is published we post the peer reviewers' comments and the authors' responses online. We also post the versions of the paper that were used during peer review. These are the versions that the peer review comments apply to.

The versions of the paper that follow are the versions that were submitted during the peer review process. They are not the versions of record or the final published versions. They should not be cited or distributed as the published version of this manuscript.

BMJ Open is an open access journal and the full, final, typeset and author-corrected version of record of the manuscript is available on our site with no access controls, subscription charges or pay-per-view fees (http://bmjopen.bmj.com).

If you have any questions on BMJ Open's open peer review process please email info.bmjopen@bmj.com

BMJ Open

Weight change, cardio-metabolic risk factors, and cardiovascular incidence in people with serious mental illness: protocol of a population-based cohort study in the UK from 1998 – 2020

Journal:	*BMJ Open*
Manuscript ID	bmjopen-2021-053427
Article Type:	Protocol
Date Submitted by the Author:	17-May-2021

Complete List of Authors:	Lee, Charlotte; University of Oxford, Nuffield Department of Primary Care Health Sciences; NIHR Oxford Biomedical Research Centre, Oxford University Hospitals NHS Foundation Trust<br>Waite, Felicity; University of Oxford, Department of Psychiatry; Oxford Health NHS Foundation Trust<br>Smith, Margaret; University of Oxford, Nuffield Department of Primary Care Health Sciences; NIHR Oxford Biomedical Research Centre<br>Gao, Min; University of Oxford, Nuffield Department of Primary Care Health Sciences; Peking University Health Science Centre, School of Public Health<br>Bankhead, Clare; University of Oxford, Nuffield Department of Primary Care Health Sciences; NIHR Oxford Biomedical Research Centre<br>Aveyard, Paul; University of Oxford, Primary Care Health Sciences; NIHR Oxford Biomedical Research Centre<br>Piernas, Carmen; University of Oxford, Nuffield Department of Primary Care Health Sciences

Keywords:	Schizophrenia & psychotic disorders < PSYCHIATRY, PRIMARY CARE, EPIDEMIOLOGY

SCHOLARONE™
Manuscripts

I, the Submitting Author has the right to grant and does grant on behalf of all authors of the Work (as defined in the below author licence), an exclusive licence and/or a non-exclusive licence for contributions from authors who are: i) UK Crown employees; ii) where BMJ has agreed a CC-BY licence shall apply, and/or iii) in accordance with the terms applicable for US Federal Government officers or employees acting as part of their official duties; on a worldwide, perpetual, irrevocable, royalty-free basis to BMJ Publishing Group Ltd ("BMJ") its licensees and where the relevant Journal is co-owned by BMJ to the co-owners of the Journal, to publish the Work in this journal and any other BMJ products and to exploit all rights, as set out in our licence.

The Submitting Author accepts and understands that any supply made under these terms is made by BMJ to the Submitting Author unless you are acting as an employee on behalf of your employer or a postgraduate student of an affiliated institution which is paying any applicable article publishing charge ("APC") for Open Access articles. Where the Submitting Author wishes to make the Work available on an Open Access basis (and intends to pay the relevant APC), the terms of reuse of such Open Access shall be governed by a Creative Commons licence – details of these licences and which Creative Commons licence will apply to this Work are set out in our licence referred to above.

Other than as permitted in any relevant BMJ Author's Self Archiving Policies, I confirm this Work has not been accepted for publication elsewhere, is not being considered for publication elsewhere and does not duplicate material already published. I confirm all authors consent to publication of this Work and authorise the granting of this licence.

For peer review only

TITLE PAGE

Weight change, cardio-metabolic risk factors, and cardiovascular incidence in people with serious mental illness: protocol of a population-based cohort study in the UK from 1998 – 2020

Authors and Affiliations:

Charlotte Lee[a,b], Felicity Waite[c,d], Margaret Smith[a,b], Min Gao[a,e], Clare Bankhead[a,b], Paul Aveyard[a,b*], Carmen Piernas[a*],

a. Nuffield Department of Primary Care Health Sciences, University of Oxford, Oxford, OX2 6GG. United Kingdom.

b. NIHR Oxford Biomedical Research Centre, Oxford University Hospitals NHS Foundation Trust.

c. Department of Psychiatry, University of Oxford, Warneford Hospital, Warneford Lane, Headington, Oxford, OX3 7JX. United Kingdom.

d. Oxford Health NHS Foundation Trust, Warneford Lane, Headington, Oxford, OX3 7JX. United Kingdom.

e. School of Public Health, Peking University Health Science Centre, Beijing, China.

*These authors equally contributed to this work.

Corresponding Author:

Charlotte Lee
E: charlotte.lee@phc.ox.ac.uk
T: +44 (0)1865 289284

Word count: 3892 (excl. title page, abstract, acknowledgments, references, and headings)

For peer review only

Abstract

Introduction: People with a serious mental illness (SMI), including schizophrenia spectrum and bipolar disorder, have increased cardiovascular disease (CVD) morbidity and mortality compared with the general population. Obesity and related co-morbidities are preventable risk factors for CVD in this population.

Aim: To improve the understanding and management of obesity and CVD in people with SMI, we will examine the prospective association between SMI status and: (1) mean weight change; (2) cardio-metabolic risk factors for CVD; and (3) incidence of CVD and CVD fatalities. We will also (4) examine the prevalence of referral to weight management services for people with SMI.

Methods and analysis: In this retrospective cohort study, we will link general practice records from the United Kingdom (UK) Clinical Practice Research Datalink (CPRD) Aurum database between 1998 and 2020. We will establish a cohort of patients with an SMI diagnosis matched with four controls without a diagnosis on age, sex, general practice, and calendar year. We will use multivariable linear regression models and Cox proportional hazard models with sequential adjustment for potential confounders identified by separate directed acyclic graphs.

Ethics and dissemination: This study has been reviewed and approved by the Independent Scientific Advisory Committee for Medicines and Healthcare products Regulatory Agency (MHRA) database research. The results will be published in a peer-reviewed journal.

Abstract word count: 215

Key words: serious mental illness, psychosis, primary care, cohort study, weight, cardiovascular

Article summary

Strengths and Limitations of this study

- Representative study population contained in the CPRD.
- Sizeable dataset and substantial statistical power.
- Potential to directly inform guidelines for the management of obesity in people with SMI.
- Observational nature study.
- Lack of information on some factors may cause residual confounding.

Introduction

People with serious mental illness (SMI), including schizophrenia and bipolar disorder, have a life expectancy that is approximately 15 to 20 years lower than the general population.[1] Although suicide is a contributing factor, approximately two-thirds of premature mortality in this group is explained by cardiovascular disease (CVD).[2] Globally, CVD is the leading cause of death claiming around 17.8 million lives.[3] But a 2017 meta-analysis reported people with SMI have a significantly increased risk of CVD

For peer review only - http://bmjopen.bmj.com/site/about/guidelines.xhtml

and CVD-related mortality than the general population: hazard ratio [HR]: 1·78, 95% confidence interval [CI]: 1·60, 1·98.[4] Other evidence suggests the mortality gap between people with SMI and the general population is growing, and indicates an important clinical need to identify the factors underlying CVD for this group.[5]

Obesity (body mass index [BMI] ≥30 kg/m^2) is a major preventable risk factor for CVD, though other factors include cardio-metabolic abnormalities like hypertension, dyslipidaemia, and insulin resistance, as well as smoking. Indeed, it is well-established that obesity is causally linked to cardio-metabolic abnormalities, and both are more common in people with SMI than the general population.[6]

The causes for the increased prevalence of obesity and CVD in people with SMI are multifactorial. Psychotropic drugs (i.e., antipsychotics, antidepressants, mood stabilisers) are the mainstay treatment for SMI, and have been linked to rapid weight gain.[7] This may contribute to CVD risk but the long-term adverse cardio-metabolic and mortality effects are not well understood.[8,9] In one 11-year follow-up study of people with schizophrenia, increased CVD mortality was reported in people treated with quetiapine (adjusted HR: 1·41, 95% CI: 1·09, 1·82), and reduced CVD mortality in people treated with clozapine (adjusted HR: 0·74, 95% CI: 0·60, 0·91). However, this study is limited by unmeasured confounding of some demographic factors.[10,11]

Unhelpful dietary patterns and physical inactivity also reflect the social disadvantage to which SMI contributes and may lead to obesity as they do for the general population. These are more apparent in people with SMI, possibly due to antipsychotic medication induced hunger and fatigue.[12] Other studies using polygenic risk scores for liability to schizophrenia find no association between those scores and physical health outcomes except for conditions mediated through obesity (and smoking).[13,14] This suggests that the risks to people with SMI are modifiable.

Currently, 73·6% of adults in the United States (US) and 62·3% of adults in the United Kingdom (UK) are classified as overweight or obese.[15] Yet, the prevalence of overweight and obesity has not been consistently reported for people with SMI. A 2018 report by Public Health England estimates people with SMI are three times more likely to have obesity for ages 15 to 34, and 1·6 times more likely for ages 55 to 74 compared with the general population.[16] But long-term data is scarce and other estimates from observational studies are often drawn from cross-sectional analyses of small numbers of people attending specific services within a geographical area with no comparison group[4].

Importantly, we have been unable to find studies that have followed people with SMI over several years to examine how weight changes and what drives those changes, including psychotropic medication or weight management interventions. The longest study to date was an 18-month trial of people with schizophrenia. In this study, participants randomised to olanzapine had greater increases in weight, glycaemia, and adverse lipid profile than other antipsychotics.[17] But randomised controlled trials (RCTs) are also limited to short follow-up, selected populations, and methodological issues surrounding imputation strategies used to address high dropout rates.[1819] To that end, we do not know the extent to which weight change explains the cardio-metabolic abnormalities and higher incidence of and mortality from CVD in people with SMI.

For peer review only - http://bmjopen.bmj.com/site/about/guidelines.xhtml

Since obesity is modifiable, US and UK clinical guidelines recommend intervening for the prevention and management of obesity. The first-line treatment is a general practitioner (GP) referral to a community weight management programme (CWMP) to support an energy-restricted diet and increase physical activity for people with a body mass index (BMI) of 25kg/m^2 or higher.[20,21] In the general population, trials of such interventions lead to greater weight-loss than self-directed weight-loss attempts.[22] But another study reported the rates of referral between 2005 and 2012 was 20·0 (95% CI: 19.3 to 20·8) per 1000 person-years, compared with weight-related advice at 30·3 (29·3 to 31·4).[23] This rate is low, and it could be that some interventions are not recorded, but other evidence also points to low rates of intervention.[24] Yet we have no data on whether GPs are using the annual review of people with SMI, which focuses specifically on preventative healthcare, to provide support for weight loss, such as by referral to CWMPs.

Primary care is suitable to monitor and opportunistically offer a referral to a weight management programme for the prevention and management of obesity and its associated risks. This is true in both the general population and people with SMI who are at even higher risk for adverse physical health. However, longitudinal data outlining weight and cardio-metabolic change, the association with incidence of and mortality from CVD, and how this is currently managed for people with SMI is lacking. In this protocol, we outline our plans to address this gap in a large, retrospective, population-based study in people with SMI matched with controls without SMI in the UK from 1998 to 2020. Our objectives are:

- To examine the prospective association between SMIs and mean weight change;
- To examine the prospective association between SMIs and change in levels of biomarkers of CVD risk;
- To examine the prospective association between SMIs and incidence of CVD and CVD fatalities;
- To compare incidence of offer and actual referral to weight management services in people with both SMIs and obesity (BMI ≥ 30 kg/m^2) with controls.

Methods and analysis

a. Patient and public involvement

We consulted 12 members of the public with lived experience of SMI to ensure the outcomes that matter to them are included in the protocol. Their continued involvement will inform the dissemination of our results.

b. Design

This protocol is written according to the ‘REporting of studies Conducted using Observational Routinely-collected health Data’ (RECORD) Statement. We will use a retrospective cohort design using individual patient data from a large primary care database and a comparison cohort to examine all objectives.

For peer review only - http://bmjopen.bmj.com/site/about/guidelines.xhtml

c. Data Source

We will use the Clinical Practice Research Datalink (CPRD) Aurum – a governmental, not-for-profit research service holding anonymised primary care records drawn from participating general (primary care) practices dating back to 1987 (http://www.cprd.com). To date, it encompasses routinely collected patient data records of around 13 million patients who are registered with some 700 participating general practices. Patient demographic data (i.e., year of birth, sex, ethnicity), characteristics (i.e., height, weight, smoking status), symptoms, clinical diagnoses, consultations, referrals, prescribed medications and results of investigations are available.

d. Data Linkage

Data will be linked to the Hospital Episode Statistics (HES) and the Office for National Statistics (ONS) mortality databases. Data linkage to the Practice Level Index of Multiple Deprivation (IMD), as a proxy measure for socio-economic status (SES), will also be used when describing the baseline characteristics in the descriptive analyses and in secondary analyses.

e. Population

Source Population:

The source population will be selected from the CPRD Aurum. The source population will include all UK National Health Service (NHS) patients (male/female) registered with a participating general practice who are eligible for data linkage. We will not include up-to-standard (UTS) records because this is not yet available as a measure of data quality in the CPRD Aurum database.

Study Cohort:

From the source population, a study cohort will be selected to investigate objectives 1 to 3. We will identify and extract the records in the primary care and HES dataset for all patients with a diagnosis of SMI (hereafter *exposed*) and who are eligible for data linkage. Diagnostic Read codes, which are the standard clinical codes used in UK general practice, will be used to identify an SMI diagnosis. We will also use codes according to the International Classification of Diseases version 10 (ICD-10) to identify indications. The date of the first SMI Read code will be used as a proxy for the date of diagnosis as well as the index date, which is the earliest date that an exposed may enter the study. From the study cohort, a sub-cohort of patients will be selected to investigate objective 4. To enter the sub-cohort, the exposed need a recorded BMI ≥ 30 kg/m^2 at any time after the index date. All information will be extracted for the period 01/01/1998 to 31/12/2020 – the earliest and latest dates respectively that data are available.

Exclusion Criteria:

Patients will be excluded from the cohort(s) if they: less than one-year of active data in their participating practice before the index date; and/or who have a Read code for coronary heart disease, congestive heart failure or cardiomyopathy, and/or stroke before the index date. A flow diagram will present the selection and exclusion (with reasons where possible) of patients in the study.

For peer review only - http://bmjopen.bmj.com/site/about/guidelines.xhtml

Duration of Follow-Up:

Patients will be followed-up in the database from their index date until the earliest of the following: date of death; date of outcome of interest; date of leaving the participating practice; date of the latest download of data; or the end of the study follow-up period.

Comparison Group:

The unexposed represents the counterfactual and will include patients without an SMI Read code ever (hereafter *unexposed*) drawn from the source population. Up to four unexposed matches will be selected at baseline only if they have a qualifying weight Read code within +/- one year of the index date to the exposed in which they are matched. The matching variables include: age, sex, practice, and calendar time. All weight Read codes will be extracted.

f. Exposures

For all analyses, the exposure of interest is SMI. We will extract information on the study cohort up to end of follow-up. Extracted information includes the diagnosis date and diagnosis type, which will be grouped according to the two major SMI sub-groups as classified by the ICD-10: F20-29 non-affective psychotic disorder (i.e., schizophrenia, schizotypal and delusional disorders, psychosis not otherwise specified [NOS]) and F30-39 affective psychotic disorder (i.e., bipolar, manic, schizoaffective disorders, major depressive disorder with psychosis). It is possible patients will have Read codes in the primary care data relating to both non-affective and affective psychotic disorders. These will be classified using the most recent Read code insofar we assume that the latest diagnosis is the correct one. Based on similar studies, instances where there are Read codes relating to both diagnoses at the same date will be coded under non-affective psychotic disorder.[25]

g. Outcomes

The primary aim is objective 1 and our outcome is weight change. We will then examine the extent to which weight change explains the prospective association between SMI status and: change in cardio-metabolic risk factors for CVD (objective 2), and incidence of CVD and CVD fatalities (objective 3). We will interpret the data based on the associations for all outcomes, as well as consistency and interpretable patterns. The specific outcomes for each objective are outlined in Table 1.

Table 1. Outcome measures

**Objective**	**Outcome**	**Measures**
Objective 1: Weight from index date to follow-up (censored date)	Weight change	Absolute mean weight change (kg)
		Percentage weight change (kg)
		BMI change
Objective 2: Biomarkers of CVD risk from index date to follow-up (censored date)	Cardio-metabolic risk	Change in mean total-cholesterol
		Change in LDL
		Change in triglycerides
		Change in FPG
		Change in HbA1c
		Change in blood pressure (systolic and diastolic)
Objective 3: Total incident CVD	Incidence of CVD and CVD	Angina diagnosed by GP
		Coronary heart disease

For peer review only - http://bmjopen.bmj.com/site/about/guidelines.xhtml

For peer review only

	fatalities	Congestive heart failure
		Cardiomyopathy
		Total stroke
		Total fatal CVD events
Objective 4: Weight management service referral	Proportion (%) of exposed and unexposed who are recorded as offered and actually referred to weight management service	

h. Confounding

We have identified confounding variables using directed acyclic graphs (DAGs), which aims to theoretically formalise potential confounders, colliders, and biasing relationships.[26] The DAG for each objective is described in the Supplementary Material S1. The DAG for objective 1 shows one direct path from the exposure (i.e., SMI status) to the outcome (i.e., weight change), and four indirect paths: via health behaviours (i.e., alcohol use, smoking, and diet), SES status, psychotropic medication, and healthcare access and provision. Diet is a latent variable in CPRD Aurum because it is unmeasured. There are two confounding paths: via age and ethnicity.[27,28] Thus, the minimally sufficient adjustment for the total effect of SMI status on weight is to adjust for age and ethnicity.

[Insert Supplementary Material S1].

We plan to address confounding by frequency matching the exposed with unexposed on age, as well as on sex, practice, and calendar year as described above. In addition, we will fully adjust our models by considering several covariates in the relationship between the exposure and outcome (see Table 2). All categorical variables will be analysed using the natural reference category as the reference group. For psychotropic medications, drugs will be classified according to the British National Formulary (BNF). Data on individual prescriptions for psychotropic medication will be extracted from the primary care data from index date until end of follow-up. For objective 3, we will examine mediation between SMI status and CVD incidence using sequential adjustment to assess the degree to which the coefficient for the association between SMI and incidence of CVD changes. For objective 4, we will assess the presence of confounding in the association between SMI status and weight loss referral by comparing estimates with and without adjustment. We will use Read codes, which have been previously developed by our team, to identify outcome and covariate records.

Table 2. Confounders

**Confounders and covariates**		**Measures**
Health Behaviours	Alcohol use	Categorical (0=low, 1=moderate, 2=high, 3=dependency). This is coded in GP records. We will use the most recent recorded value of alcohol use at the study start date.
	Smoking status	Categorical (0=never; 1=ex-smoker; 2=current smoker [light: 1-9 cigarettes/day; moderate: 10-19 cigarettes/day; heavy: 20+ cigarettes/day]). This is coded in GP records. We will use the most recent recorded value of smoking status at the study start date.

For peer review only - http://bmjopen.bmj.com/site/about/guidelines.xhtml

Demographics	Socioeconomic status (SES)	Categorical; Practice Level Index of Multiple Deprivation (IMD), an indicator of material deprivation (quintiles)
	Ethnicity	Categorical (White, Indian, Pakistani, Bangladeshi, other Asian, Black African, Chinese, and other ethnic group)
	Sex	Categorical
	Age at study entry, in years	Continuous
Physical and/or medical factors	Weight measured as baseline body mass index (BMI)	Categorical (18.5-24.9 [normal weight], 25.0-29.9 [overweight)], 30.0-34.9 [obese], 35.0-35.9 [severe obesity], 40.0-44.9 kg/m^2 [morbid obesity)]).
	Psychotropic medication	Categorical
We include the following additional variables for objectives 3 and 4 only		
Physical and/or medical factors	High cholesterol	Binary (0=no; 1=yes)
	Hypertension	Binary (0=no; 1=yes)
	Diabetes	Binary (0=no; 1=yes)
	Presence of all risk factors	Binary (0=no; 1=yes)

i. Variable Library

Exposures, outcomes, and covariates of interest will be identified using medical coding (Read/SNOMED/EMIS), product coding (DM+D), and recording of patient death or transfer out of a participating practice in CPRD Aurum. The final definitions and code library has been drawn up and validated through consensus agreement amongst all investigators.

j. Sample Size Calculation

This cohort study will include all available adults with a diagnosis of SMI meaning the size of the sample ultimately depends on the number of patients in the dataset with a recorded SMI diagnosis. Hence, we present a basic sample calculation using parameter estimates obtained from previous studies and based on standard formulae. For the purpose of this calculation, we estimated a follow-up time for the exposed with a weight Read code in their record in the one-year *before* the index date. The estimated median follow-up time for the exposed was 3.2 years (mean: 4.6). We assumed a 1% annual incidence rate of CVD meaning 3% of the study population would develop CVD over the ~3-years of follow-up estimated above.[29] We also assumed a relative risk (RR) of 1.3 for CVD in the exposed versus unexposed.[30] The *n* ratio was set at 0.25 (*exposed:unexposed* ratio 1:4), which achieves the best balance between data cost and statistical power. Our calculation suggested a minimum sample size of *n*=3,907 to be 80% powered to detect a RR of 1.3, alpha $\leq$0.05%. The estimated number of patients in the CPRD Aurum is n~135,870.

k. Statistical analysis

Analyses will be conducted in Stata using an estimated approach (alpha $\leq$0 · 05 two-tailed). We do not intend to impute missing data. The entry date into the analysis is the index date. The outcome date is the earliest of: (i) the date of death, or (ii) date of diagnosis of the outcome of interest. For objectives 1 and 2, we will use all repeated measures and run separate models for each analyses. For objective

For peer review only - http://bmjopen.bmj.com/site/about/guidelines.xhtml

3, we will use the first recorded diagnosis of the outcome of interest rather than recurrent events. Patients who do not have the outcome of interest will be censored at the earliest of: (i) date of death, (ii) date of leaving the practice, (iii) date of the latest download of data, or (iv) the study end date.

Descriptive summaries:

Frequency matching will be done to reduce potential confounding and to balance the baseline characteristics of the exposed and unexposed on age, sex, practice, and study year. Adequacy of matching will be confirmed by comparing means using independent *t*-tests. Descriptive statistics will describe the baseline characteristics of patients in the cohort. Categorical data will be presented as frequency and percentages, and continuous variables will be summarised using descriptive statistics (mean, standard deviation [SD], or median and interquartile range). Comparisons between the groups will be made using χ^2 tests and *t*-tests (or Wilcoxon rank-sum tests) as appropriate. Length of exposure and the most commonly prescribed class of psychotropic for each patient will be summarised. The causes of death will be categorised as CVD and all-cause mortality.

Primary analyses:

For objectives 1 and 2, we will use a mixed-linear regression model examining SMI x time and psychotropic medications x time to estimate regression coefficients for weight change and cardio-metabolic risk with 95% CIs in the total sample and by SMI status. We will include a random effect for practice identifier to account for intra-class correlation (ICC) resulting from common practice membership. We will add interactions and, if significant, we will assess the strength of the associations in separate sub-groups for clarity. For objective 3, we will use Kaplan-Meier survival estimates for the exposed and unexposed groups to estimate the distribution of time from index date to total and fatal CVD risk and mortality (all-cause). The statistical equivalence of the two curves will be examined using the log-rank test. This analysis will consider survival from index date between the exposed and unexposed groups using an adjusted Cox proportional hazards regression model with follow-up time as the timescale. For objective 4, we will use a Poisson regression examining person-time to estimate the proportion of patients who received weight management interventions over the study period by diagnosis and BMI category and to investigate variables associated with referral.

Sensitivity and secondary analyses:

If necessary, data on missing confounders and covariates will be addressed by adjusting for missingness as a category. Sensitivity analyses will examine the impact of running the analyses with everyone included as discussed above and confined only to those with complete data. A second sensitivity analysis will be performed excluding patients that experience the outcome within two years of the SMI Read code to avoid reversed causality from other underlying disease.

Discussion

In the UK, the NHS Long Term Plan commits the health service to greater action on reducing inequalities and narrowing variation in health outcomes for marginalised and the most vulnerable groups. The plan sets out specific actions, for example, by reducing obesity in people given mental health diagnoses through physical health checks and upstream prevention. The plan also commits the NHS to support people with SMI in greater choice and control over their healthcare.

For peer review only - http://bmjopen.bmj.com/site/about/guidelines.xhtml

This study responds to this national agenda by leveraging the power of a large UK primary care database to provide new insight on how these actions may be achieved. Our results will estimate the long-term likelihood of weight gain in people with SMI, which could inform medication choice for both people with SMI and clinicians, particularly if there are meaningful differences between psychotropic medications (objective 1). Assessing the incidence of cardio-metabolic factors (i.e., hypertension, adverse lipid profiles, and glycaemia) relative to those without SMI will be valuable because these are key health outcomes that are being addressed (objective 2). Assessing whether the increased risk in CVD is due to changes in cardio-metabolic factors will help us understand the degree to which these are valuable therapeutic targets (objective 3). Understanding whether this group are being offered support to lose weight informs national initiatives to overcome therapeutic nihilism. It is also important to understand whether there are differences in response between people with SMI and the general population, which would inform us about whether brief interventions need to be tailored to people with SMI for more personalised care (objective 4). Ultimately, the results will offer a foundation to assess the degree to which the current focus on CVD prevention in people with SMI is having clinical benefits.

Since this study is observational, there are inherent strengths and limitations that are discussed. A strength of the study is its use of a high-quality, validated, sizeable dataset. The CPRD Aurum is an appropriate data source utilising primary care records, which minimises selection, recall, and respondent bias since UK general practices tend to have good levels of accuracy in recording prescriptions and clinical diagnoses. The results will also have direct and important applications to international guidelines on patient healthcare management, as well as to British primary care physicians, mental health services, and members of the public with experience of SMIs to initiate individual action. Furthermore, the longitudinal design is useful in addressing important clinical questions not usually possible within RCTs, and a matched-cohort improves efficiency since we will not need to include all controls, whilst tackling confounding.

Since the study is descriptive, any association observed would not imply an SMI diagnosis is causative of adverse physical health outcomes. However, we might assume the greater the magnitude of the observed association the less likely it is due to bias and more likely due to a causal process[33]. Unmeasured confounders, including poverty, may place people with SMI at increased risk of homelessness, which has consequences on access and provision of healthcare. We address this potential confounding by linking to Patient Level IMD as a proxy measure of SES, but acknowledge this may not fully capture deprivation and leaves room for residual confounding. Information bias may be present since the CPRD Aurum relies on a GP recording of health outcomes. Furthermore, we can only examine primary care prescriptions. Hence, any prescribing solely within secondary care may not be included as this was not historically routinely recorded in GP records. Thus, it is also possible we will miss the first prescription. Physical health checks are not always performed on random samples of the population, and it is possible that regular testing is done on people who are overweight, and at risk of type 2 diabetes and CVD. Similarly, it is also expected that some patients will have incomplete information recorded. By determining what proportion of patients do not have appropriate information recorded, we can evaluate the scale of these problems, and highlight the need for improved recording for continuity of care between healthcare professionals.

For peer review only - http://bmjopen.bmj.com/site/about/guidelines.xhtml

Author Statement

Acknowledgements

We thank the patient and public Involvement volunteers for their time and for sharing their knowledge. We also thank the patients and practices who contribute to the CPRD database. We acknowledge that HES data that will be used in this analysis will be re-used with permission from NHS Digital, who retain the copyright.

Ethics and dissemination

This study has been approved by the Independent Scientific Advisory Committee (ISAC) to the Medicines and Healthcare Products Regulatory Agency (reference: 20_000186). To guarantee the confidentiality of patient information, only the authors will have access to the data during the study. The full statistical code will be available from the authors after the publication of the results.

Author contributions

CL, CPS, FW, and PA conceived the study idea. All authors contributed to the development of the study design and protocol. CL drafted the manuscript. All authors approved the final submitted version.

Funding

This research received no specific grant from any funding agency in the public, commercial or not-for-profit sectors. CL is funded by the Engineering and Physical Sciences Research Council (EPSRC; EP/R513295/1) and the National Institute for Health Research (NIHR) Oxford Biomedical Research Centre (BRC; IS-BRC-1215-20008). MS is supported by the NIHR Oxford BRC. CB is supported by the NIHR Oxford BRC. PA is funded by the NIHR BRC, the NIHR ARC (200172), and is an NIHR senior investigator (NF-SI-0617-10064). CPS is funded through the NIHR ARC. FW is funded by a Wellcome Trust Clinical Doctoral Fellowship (102176/B/13/Z).

Disclaimer

The views expressed are those of the author(s) and not necessarily those of the NIHR or the Department of Health and Social Care.

Conflicts of interests

PA has conducted a trial funded by Cambridge Weight Plan, and has spoken at a seminar at the Royal College of General Practitioners (RCGP) conference that was sponsored by Novo Nordisk. None of these activates led to personal payments to him. All other authors declare no conflict of interests.

Patient consent for publication

Not required.

Data statement

The data was made available by the CPRD obtained under licence from the UK MRHA. Data is available to researchers who are approved by the Independent Scientific Advisory Committee.

References

1. Hennekens CH, Hennekens AR, Hollar D, Casey DE. Schizophrenia and increased risks of

For peer review only - http://bmjopen.bmj.com/site/about/guidelines.xhtml

cardiovascular disease. *Am Heart J*. 2005;150(6):1115-1121. doi:10.1016/j.ahj.2005.02.007
2. Osborn DPJ, Levy G, Nazareth I, Petersen I, Islam A, King MB. Relative risk of cardiovascular and cancer mortality in people with severe mental illness from the United Kingdom's General Practice Research Database. *Arch Gen Psychiatry*. 2007;64(2):242-249. doi:10.1001/archpsyc.64.2.242
3. Yusuf S, Reddy S, Ôunpuu S, Anand S. Global Burden of Cardiovascular Diseases. *Circulation*. 2001;104(22):2746-2753. doi:10.1161/hc4601.099487
4. Correll CU, Solmi M, Veronese N, et al. Prevalence, incidence and mortality from cardiovascular disease in patients with pooled and specific severe mental illness: a large-scale meta-analysis of 3,211,768 patients and 113,383,368 controls. *World Psychiatry*. 2017;16(2):163-180. doi:10.1002/wps.20420
5. Saha S, Chant D, McGrath J. A systematic review of mortality in schizophrenia: Is the differential mortality gap worsening over time? *Arch Gen Psychiatry*. 2007;64(10):1123-1131. doi:10.1001/archpsyc.64.10.1123
6. Mitchell AJ, Vancampfort D, Sweers K, Van Winkel R, Yu W, De Hert M. Prevalence of metabolic syndrome and metabolic abnormalities in schizophrenia and related disorders-a systematic review and meta-analysis. *Schizophr Bull*. 2013;39(2):306-318. doi:10.1093/schbul/sbr148
7. Leslie WS, Hankey CR, Lean MEJ. Review Weight gain as an adverse effect of some commonly prescribed drugs: a systematic review. *QJM*. 2007;100:395–404. doi:10.1093/qjmed/hcm044
8. De Hert M, Schreurs V, Sweers K, et al. Typical and atypical antipsychotics differentially affect long-term incidence rates of the metabolic syndrome in first-episode patients with schizophrenia: A retrospective chart review. *Schizophr Res*. 2008;101(1-3):295-303. doi:10.1016/j.schres.2008.01.028
9. Pillinger T, McCutcheon RA, Vano L, et al. Comparative effects of 18 antipsychotics on metabolic function in patients with schizophrenia, predictors of metabolic dysregulation, and association with psychopathology: a systematic review and network meta-analysis. *The Lancet Psychiatry*. 2020;7(1):64-77. doi:10.1016/S2215-0366(19)30416-X
10. Tiihonen J, Lönnqvist J, Wahlbeck K, et al. 11-year follow-up of mortality in patients with schizophrenia: a population-based cohort study (FIN11 study). *Lancet*. 2009;374(9690):620-627. doi:10.1016/S0140-6736(09)60742-X
11. De Hert M, Correll CU, Cohen D. Do antipsychotic medications reduce or increase mortality in schizophrenia? A critical appraisal of the FIN-11 study. *Schizophr Res*. 2010;117(1):68-74. doi:10.1016/j.schres.2009.12.029
12. Teasdale SB, Ward PB, Samaras K, et al. Dietary intake of people with severe mental illness: Systematic review and meta-analysis. *Br J Psychiatry*. 2019;214(5):251-259. doi:10.1192/bjp.2019.20
13. Holt RIG, Peveler RC. Obesity, serious mental illness and antipsychotic drugs. *Diabetes, Obes Metab*. 2009;11(7):665-679. doi:10.1111/j.1463-1326.2009.01038.x
14. Kendall KM, John A, Lee SC, et al. Impact of schizophrenia genetic liability on the association between schizophrenia and physical illness: data-linkage study. *BJPsych Open*. 2020;6(6). doi:10.1192/bjo.2020.42
15. NHS Digital. QOF 2016/17 results. https://qof.digital.nhs.uk/. Published 2019. Accessed November 14, 2019.
16. National Mental Health Intelligence Network. Severe mental illness (SMI) and physical health inequalities: briefing - GOV.UK. Public Health England. https://www.gov.uk/government/publications/severe-mental-illness-smi-physical-health-inequalities/severe-mental-illness-and-physical-health-inequalities-briefing#fn:1. Published 2018. Accessed November 14, 2019.
17. Lieberman JA, Stroup TS, McEvoy JP, et al. Effectiveness of Antipsychotic Drugs in Patients with Chronic Schizophrenia. *N Engl J Med*. 2005;353(12):1209-1223.

For peer review only - http://bmjopen.bmj.com/site/about/guidelines.xhtml

doi:10.1056/nejmoa051688
18. Jones PB, Barnes TRE, Davies L, et al. Randomized controlled trial of the effect on quality of life of second- vs first-generation antipsychotic drugs in schizophrenia: Cost Utility of the Latest Antipsychotic Drugs in Schizophrenia Study (CUtLASS 1). *Arch Gen Psychiatry*. 2006;63(10):1079-1087. doi:10.1001/archpsyc.63.10.1079
19. Álvarez-Jiménez M, González-Blanch C, Crespo-Facorro B, et al. Antipsychotic-induced weight gain in chronic and first-episode psychotic disorders: A systematic critical reappraisal. *CNS Drugs*. 2008;22(7):547-562. doi:10.2165/00023210-200822070-00002
20. Agency for Healthcare Research and Quality. U.S. Preventive Services Task Force. Rockville, MD. https://www.ahrq.gov/cpi/about/otherwebsites/uspstf/index.html. Published 2019.
21. The National Institute for Health and Care Excellence (NICE). Obesity prevention clinical guideline [CG43]. United Kingdom. https://www.nice.org.uk/guidance/CG43. Published 2015.
22. Jolly K, Lewis A, Beach J, et al. Comparison of range of commercial or primary care led weight reduction programmes with minimal intervention control for weight loss in obesity: Lighten Up randomised controlled trial. *BMJ*. 2011;343(7832):1035. doi:10.1136/bmj.d6500
23. Booth HP, Prevost AT, Gulliford MC. Access to weight reduction interventions for overweight and obese patients in UK primary care: Population-based cohort study. *BMJ Open*. 2015;5(1):e006642. doi:10.1136/bmjopen-2014-006642
24. Humphreys K, Blodgett JC, Roberts LW. The exclusion of people with psychiatric disorders from medical research. *J Psychiatr Res*. 2015;70:28-32. doi:10.1016/j.jpsychires.2015.08.005
25. Hope H, Parisi R, Ashcroft DM, et al. Fertility trends of women with serious mental illness in the United Kingdom 1992–2017: A primary care cohort study using the clinical practice research datalink. *J Affect Disord*. 2020;269:141-147. doi:10.1016/j.jad.2020.03.037
26. Shrier I, Platt RW. Reducing bias through directed acyclic graphs. *BMC Med Res Methodol*. 2008;8:70. doi:10.1186/1471-2288-8-70
27. Gogtay N, Vyas NS, Testa R, Wood SJ, Pantelis C. Age of onset of schizophrenia: Perspectives from structural neuroimaging studies. *Schizophr Bull*. 2011;37(3):504-513. doi:10.1093/schbul/sbr030
28. Schwartz RC. Racial disparities in psychotic disorder diagnosis: A review of empirical literature. *World J Psychiatry*. 2014;4(4):133. doi:10.5498/wjp.v4.i4.133
29. Fan Z, Wu Y, Shen J, Ji T, Zhan R. Schizophrenia and the risk of cardiovascular diseases: A meta-analysis of thirteen cohort studies. *J Psychiatr Res*. 2013;47(11):1549-1556. doi:10.1016/j.jpsychires.2013.07.011
30. Cunningham R, Poppe K, Peterson D, Every-Palmer S, Soosay I, Jackson R. Prediction of cardiovascular disease risk among people with severe mental illness: A cohort study. *PLoS One*. 2019;14(9). doi:10.1371/journal.pone.0221521
31. James Lind Alliance. Schizophrenia Top 10. https://www.jla.nihr.ac.uk/priority-setting-partnerships/schizophrenia/top-10-priorities/. Published 2021. Accessed February 12, 2021.
32. The National Institute for Health and Care Excellence (NICE). 1 Recommendations | Psychosis and schizophrenia in adults: prevention and management | Guidance | NICE. https://www.nice.org.uk/guidance/cg178/chapter/1-recommendations. Published 2014. Accessed March 2, 2021.
33. Grimes DA, Schulz KF. Bias and causal associations in observational research. *Lancet*. 2002;359(9302):248-252. doi:10.1016/S0140-6736(02)07451-2

For peer review only - http://bmjopen.bmj.com/site/about/guidelines.xhtml

Weight change, cardio-metabolic risk factors, and cardiovascular incidence in people with serious mental illness: protocol of a population-based cohort study in the UK from 1997 – 2020

Supplementary Material

Supplementary material S1. Direct acyclic graphs (DAGs) for the prospective association between SMI status and each outcome.

In the following figures, the green box (i.e., SMI status) is the exposure variable. The blue box 'I' is the outcome variable. The grey box 'diet' is a latent variable because it is unobserved in the CPRD Aurum dataset. Causal paths are indicated via green arrows; biasing paths in red; and paths with no influence on the outcome in black.

Figure 1. DAG for the prospective association between SMI status and mean weight change (objective 1).

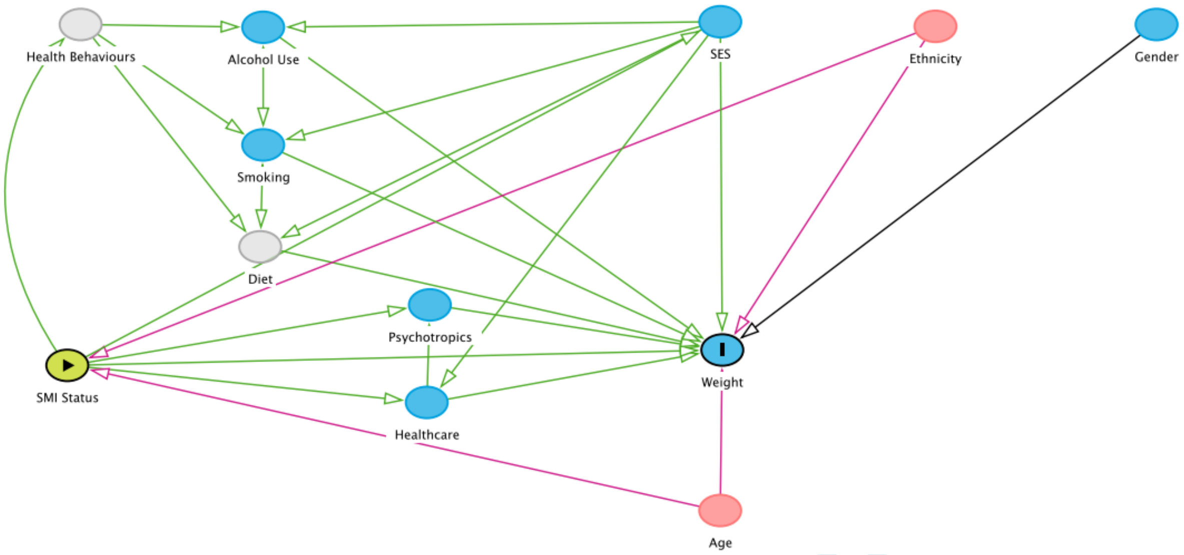

This DAG shows **five** paths between the exposure (i.e., SMI status) and outcome (i.e., weight): the (1) direct path from SMI status to weight; the indirect paths via (2) health behaviours (i.e. alcohol use, smoking, and diet), via (3) socio-economic status (SES), via (4) psychotropic medications, and via (5) healthcare access and provision. All variables on the indirect paths are mediating. There are two confounding paths: the path via age, and via ethnicity. Thus, the minimally sufficient adjustment to get the total effect of the exposure on the outcome is to adjust for age and ethnicity. The direct effect cannot be estimated by covariate adjustment.

Figure 2. DAG for the prospective association between SMI status and biomarkers for CVD risk (objective 2).

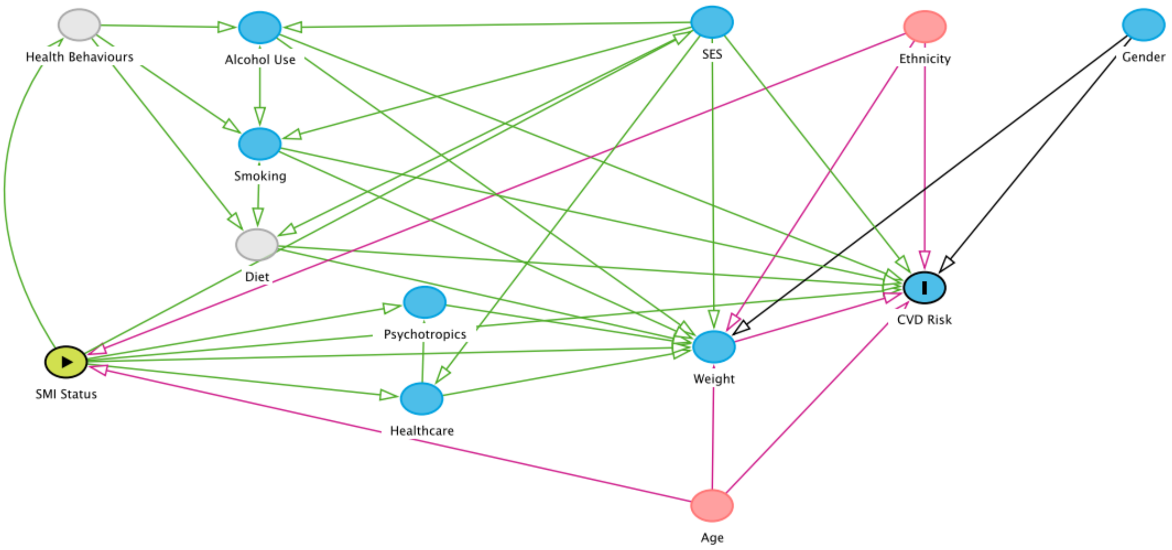

This DAG shows **six** paths between the exposure (i.e., SMI status) and outcome (i.e., CVD risk): the (1) direct path from SMI status to CVD risk; the indirect paths via (2) health behaviours (i.e. alcohol use, smoking, and diet), via (3) socio-economic status (SES), via (4) psychotropic medications, via (5) healthcare access and provision, and via (6) weight. There are two confounding paths: the path via age, and via ethnicity. Thus, the minimally sufficient adjustment to get the total effect of the exposure on the outcome is to adjust for age and ethnicity. The direct effect cannot be estimated by covariate adjustment.

For peer review only

Figure 3. DAG for the prospective association between SMI status and CVD incidence (objective 3).

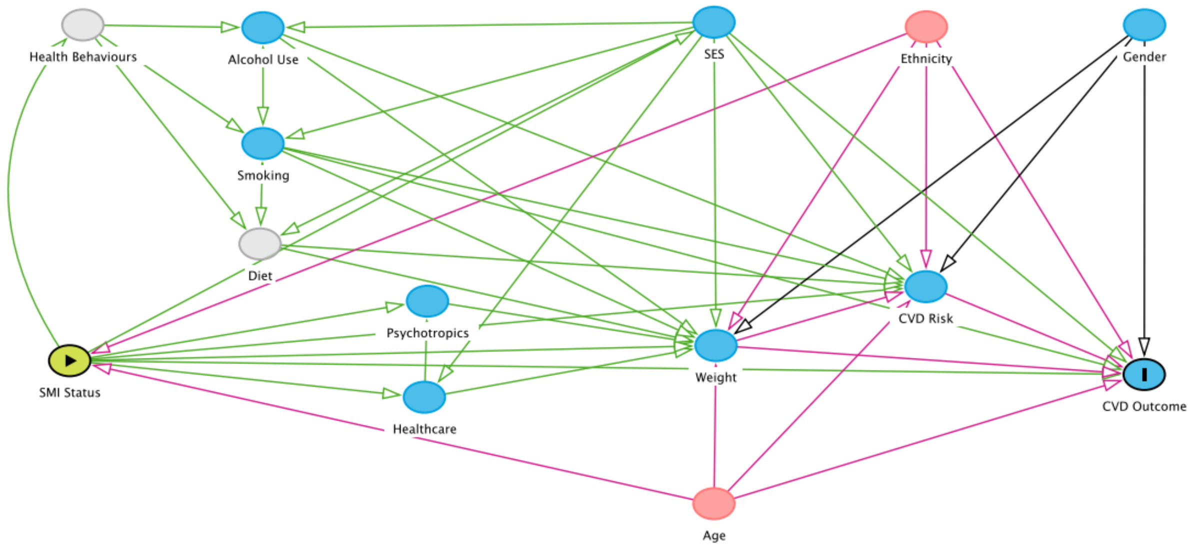

This DAG shows **seven** paths between the exposure (i.e., SMI status) and outcome (i.e., CVD risk): the (1) direct path from SMI status to CVD risk; the indirect paths via (2) health behaviours (i.e. alcohol use, smoking, and diet), via (3) socio-economic status (SES), via (4) psychotropic medications, via (5) healthcare access and provision, via (6) weight, and via (7) CVD risk. The minimally sufficient adjustment to get the total effect of the exposure on the outcome is to adjust for age and ethnicity. The minimally sufficient adjustment for estimating the direct effect of the exposure on the outcome is to adjust for: age, ethnicity, gender, SES, smoking, weight, and CVD risk.

Figure 4. DAG to compare the incidence of offer of and actual referral to weight management services in people with both a SMI and obesity and healthy controls (objective 4).

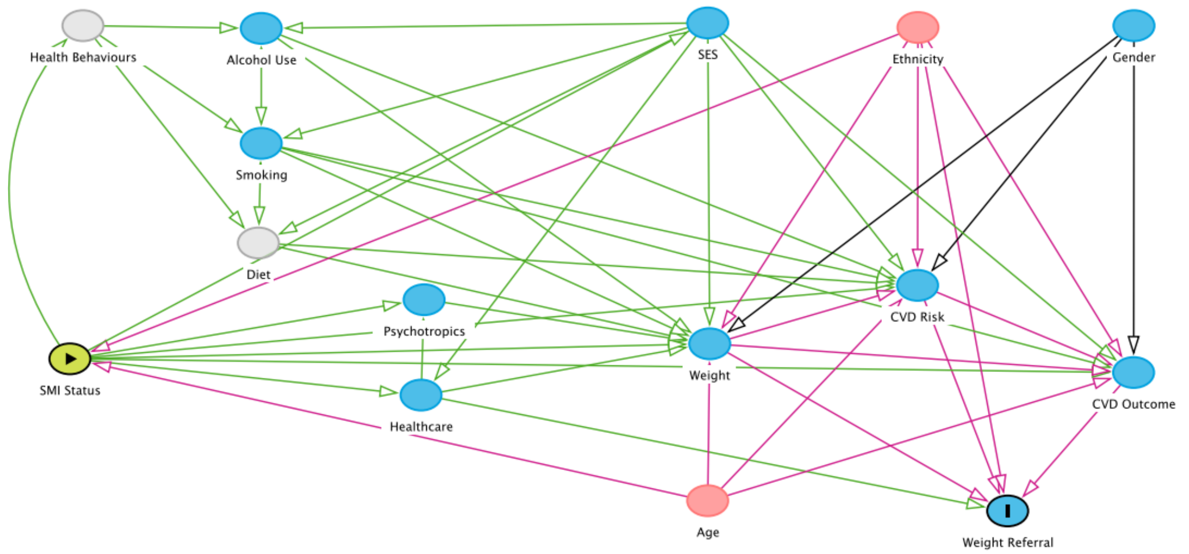

This model does not change the adjustment set.

For peer review only

The RECORD statement – checklist of items, extended from the STROBE statement, that should be reported in observational studies using routinely collected health data.

	Item No.	STROBE items	Location in manuscript where items are reported	RECORD items	Location in manuscript where items are reported
**Title and abstract**					
	1	(a) Indicate the study's design with a commonly used term in the title or the abstract (b) Provide in the abstract an informative and balanced summary of what was done and what was found	(a): Page 1 – Title; Page 2 – Abstract<br><br>(b): Not applicable because this manuscript reports a protocol	RECORD 1.1: The type of data used should be specified in the title or abstract. When possible, the name of the databases used should be included.<br><br>RECORD 1.2: If applicable, the geographic region and timeframe within which the study took place should be reported in the title or abstract.<br><br>RECORD 1.3: If linkage between databases was conducted for the study, this should be clearly stated in the title or abstract.	(1.1): Page 2 - Abstract<br><br>(1.2): Page 2 - Abstract<br><br>(1.3): Page 2 - Abstract
**Introduction**					
Background rationale	2	Explain the scientific background and rationale for the investigation being reported	Page 3 – Background		
Objectives	3	State specific objectives, including any prespecified hypotheses	Page 4 – Background		
**Methods**					
Study Design	4	Present key elements of study design early in the paper	Methods and analysis:<br>• Page 4 - Design		
Setting	5	Describe the setting, locations, and relevant dates, including periods of recruitment, exposure, follow-up, and data collection	Methods and analysis:<br>• Settings, locations, data collection: Page 4 - Data Source<br>• Periods of recruitment: Page 5 - Study Cohort<br>• Exposure: Page 6 - Exposures<br>• Follow-up: Page 5 –		

For peer review only

			Duration of Follow-Up		
Participants	6	*(a) Cohort study* - Give the eligibility criteria, and the sources and methods of selection of participants. Describe methods of follow-up<br>*Case-control study* - Give the eligibility criteria, and the sources and methods of case ascertainment and control selection. Give the rationale for the choice of cases and controls<br>*Cross-sectional study* - Give the eligibility criteria, and the sources and methods of selection of participants<br><br>*(b) Cohort study* - For matched studies, give matching criteria and number of exposed and unexposed<br>*Case-control study* - For matched studies, give matching criteria and the number of controls per case	(a): Page 5 – Study Cohort<br><br>(b): Page 5 – Comparison Group	RECORD 6.1: The methods of study population selection (such as codes or algorithms used to identify subjects) should be listed in detail. If this is not possible, an explanation should be provided.<br><br>RECORD 6.2: Any validation studies of the codes or algorithms used to select the population should be referenced. If validation was conducted for this study and not published elsewhere, detailed methods and results should be provided.<br><br>RECORD 6.3: If the study involved linkage of databases, consider use of a flow diagram or other graphical display to demonstrate the data linkage process, including the number of individuals with linked data at each stage.	(6.1): Page 5 – Study Cohort<br><br>(6.2): Not done because this manuscript reports a protocol. We will do this for any output papers.<br><br>(6.3): Considered but not thought necessary.
Variables	7	Clearly define all outcomes, exposures, predictors, potential confounders, and effect modifiers. Give diagnostic criteria, if applicable.	Methods and analysis:<br>• Exposures: Page 6 – Exposures<br>• Potential confounders and effect modifiers – Page 7 – Confounding	RECORD 7.1: A complete list of codes and algorithms used to classify exposures, outcomes, confounders, and effect modifiers should be provided. If these cannot be reported, an explanation should be provided.	(7.1): Not provided because this manuscript reports a protocol. We will provide this for any output papers.
Data sources/ measurement	8	For each variable of interest, give sources of data and details of methods of assessment (measurement). Describe comparability of assessment methods if there is more than one group	Not considered		
Bias	9	Describe any efforts to address potential sources of bias	Page 7 - Confounding		
Study size	10	Explain how the study size was arrived at	Page 8 – Sample size calculation		
Quantitative variables	11	Explain how quantitative variables were handled in the analyses. If	Page 9 – Primary analyses		

For peer review only

		applicable, describe which groupings were chosen, and why			
Statistical methods	12	(a) Describe all statistical methods, including those used to control for confounding<br>(b) Describe any methods used to examine subgroups and interactions<br>(c) Explain how missing data were addressed<br>(d) *Cohort study* - If applicable, explain how loss to follow-up was addressed<br>*Case-control study* - If applicable, explain how matching of cases and controls was addressed<br>*Cross-sectional study* - If applicable, describe analytical methods taking account of sampling strategy<br>(e) Describe any sensitivity analyses	(a): Page 9 – Statistical methods (primary analyses)<br><br>(b): Page 9 – Statistical methods (primary analyses)<br><br>(b): Page 9 – Statistical methods (sensitivity and secondary analyses)		
Data access and cleaning methods		..		RECORD 12.1: Authors should describe the extent to which the investigators had access to the database population used to create the study population.<br><br>RECORD 12.2: Authors should provide information on the data cleaning methods used in the study.	(12.1): Not considered in the manuscript. But bull access will available.<br><br>(12.2): Not yet performed.
Linkage		..		RECORD 12.3: State whether the study included person-level, institutional-level, or other data linkage across two or more databases. The methods of linkage and methods of linkage quality evaluation should be provided.	(12.3): Page 5 – Data Linkage
**Results**					
Participants	13	(a) Report the numbers of individuals at each stage of the study (*e.g.*, numbers potentially eligible, examined for eligibility, confirmed eligible, included in the study, completing follow-up, and analysed)<br>(b) Give reasons for non-participation at each stage.<br>(c) Consider use of a flow diagram	(a-c): Not applicable	RECORD 13.1: Describe in detail the selection of the persons included in the study (*i.e.*, study population selection) including filtering based on data quality, data availability and linkage. The selection of included persons can be described in the text and/or by means of the study flow diagram.	(13.1): Not applicable
Descriptive data	14	(a) Give characteristics of study participants (*e.g.*, demographic, clinical, social) and information on exposures and potential confounders	(a-c): Not applicable		

For peer review only - http://bmjopen.bmj.com/site/about/guidelines.xhtml

		(b) Indicate the number of participants with missing data for each variable of interest<br>(c) *Cohort study* - summarise follow-up time (*e.g.*, average and total amount)			
Outcome data	15	*Cohort study* - Report numbers of outcome events or summary measures over time<br>*Case-control study* - Report numbers in each exposure category, or summary measures of exposure<br>*Cross-sectional study* - Report numbers of outcome events or summary measures	Not applicable		
Main results	16	(a) Give unadjusted estimates and, if applicable, confounder-adjusted estimates and their precision (e.g., 95% confidence interval). Make clear which confounders were adjusted for and why they were included<br>(b) Report category boundaries when continuous variables were categorized<br>(c) If relevant, consider translating estimates of relative risk into absolute risk for a meaningful time period	(a-c): Not applicable		
Other analyses	17	Report other analyses done—e.g., analyses of subgroups and interactions, and sensitivity analyses	Not applicable		
Key results	18	Summarise key results with reference to study objectives	Not applicable		
Limitations	19	Discuss limitations of the study, taking into account sources of potential bias or imprecision. Discuss both direction and magnitude of any potential bias	Page 9-10 - Discussion	RECORD 19.1: Discuss the implications of using data that were not created or collected to answer the specific research question(s). Include discussion of misclassification bias, unmeasured confounding, missing data, and changing eligibility over time, as they pertain to the study being reported.	Not applicable
Interpretation	20	Give a cautious overall interpretation of results considering objectives, limitations, multiplicity of analyses, results from similar studies, and other relevant evidence	Not applicable		
Generalisability	21	Discuss the generalisability (external validity) of the study results	Page 9-10 - Discussion		
**Other Information**					

For peer review only

Funding	22	Give the source of funding and the role of the funders for the present study and, if applicable, for the original study on which the present article is based	Page 10 – Acknowledgements (Funding)		
Accessibility of protocol, raw data, and programming code		..		RECORD 22.1: Authors should provide information on how to access any supplemental information such as the study protocol, raw data, or programming code.	Page 10 – Acknowledgements (Ethics and dissemination)

*Reference: Benchimol EI, Smeeth L, Guttmann A, Harron K, Moher D, Petersen I, Sørensen HT, von Elm E, Langan SM, the RECORD Working Committee. The REporting of studies Conducted using Observational Routinely-collected health Data (RECORD) Statement. *PLoS Medicine* 2015; in press.

*Checklist is protected under Creative Commons Attribution (CC BY) license.

For peer review only

BMJ Open

Weight change, cardio-metabolic risk factors, and cardiovascular incidence in people with serious mental illness: protocol of a population-based cohort study in the UK from 1998 to 2020

Journal:	*BMJ Open*
Manuscript ID	bmjopen-2021-053427.R1
Article Type:	Protocol
Date Submitted by the Author:	26-Aug-2021

Complete List of Authors:	Lee, Charlotte; University of Oxford, Nuffield Department of Primary Care Health Sciences; NIHR Oxford Biomedical Research Centre, Oxford University Hospitals NHS Foundation Trust<br>Waite, Felicity; University of Oxford, Department of Psychiatry; Oxford Health NHS Foundation Trust<br>Smith, Margaret; University of Oxford, Nuffield Department of Primary Care Health Sciences; NIHR Oxford Biomedical Research Centre<br>Gao, Min; University of Oxford, Nuffield Department of Primary Care Health Sciences; Peking University Health Science Centre, School of Public Health<br>Bankhead, Clare; University of Oxford, Nuffield Department of Primary Care Health Sciences; NIHR Oxford Biomedical Research Centre<br>Aveyard, Paul; University of Oxford, Primary Care Health Sciences; NIHR Oxford Biomedical Research Centre<br>Piernas, Carmen; University of Oxford, Nuffield Department of Primary Care Health Sciences

**Primary Subject Heading**:	Epidemiology
Secondary Subject Heading:	Mental health
Keywords:	Schizophrenia & psychotic disorders < PSYCHIATRY, PRIMARY CARE, EPIDEMIOLOGY

SCHOLARONE™ Manuscripts

I, the Submitting Author has the right to grant and does grant on behalf of all authors of the Work (as defined in the below author licence), an exclusive licence and/or a non-exclusive licence for contributions from authors who are: i) UK Crown employees; ii) where BMJ has agreed a CC-BY licence shall apply, and/or iii) in accordance with the terms applicable for US Federal Government officers or employees acting as part of their official duties; on a worldwide, perpetual, irrevocable, royalty-free basis to BMJ Publishing Group Ltd ("BMJ") its licensees and where the relevant Journal is co-owned by BMJ to the co-owners of the Journal, to publish the Work in this journal and any other BMJ products and to exploit all rights, as set out in our licence.

The Submitting Author accepts and understands that any supply made under these terms is made by BMJ to the Submitting Author unless you are acting as an employee on behalf of your employer or a postgraduate student of an affiliated institution which is paying any applicable article publishing charge ("APC") for Open Access articles. Where the Submitting Author wishes to make the Work available on an Open Access basis (and intends to pay the relevant APC), the terms of reuse of such Open Access shall be governed by a Creative Commons licence – details of these licences and which Creative Commons licence will apply to this Work are set out in our licence referred to above.

Other than as permitted in any relevant BMJ Author's Self Archiving Policies, I confirm this Work has not been accepted for publication elsewhere, is not being considered for publication elsewhere and does not duplicate material already published. I confirm all authors consent to publication of this Work and authorise the granting of this licence.

For peer review only

TITLE PAGE

Weight change, cardio-metabolic risk factors, and cardiovascular incidence in people with serious mental illness: protocol of a population-based cohort study in the UK from 1998 to 2020

Authors and Affiliations:

Charlotte Lee[a,b], Felicity Waite[c,d], Margaret Smith[a,b], Min Gao[a,e], Clare Bankhead[a,b], Paul Aveyard[a,b*], Carmen Piernas[a*]

a. Nuffield Department of Primary Care Health Sciences, University of Oxford, Oxford, OX2 6GG. United Kingdom.

b. NIHR Oxford Biomedical Research Centre, Oxford University Hospitals NHS Foundation Trust.

c. Department of Psychiatry, University of Oxford, Warneford Hospital, Warneford Lane, Headington, Oxford, OX3 7JX. United Kingdom.

d. Oxford Health NHS Foundation Trust, Warneford Lane, Headington, Oxford, OX3 7JX. United Kingdom.

e. School of Public Health, Peking University Health Science Centre, Beijing, China.

*These authors equally contributed to this work.

Corresponding Author:

Charlotte Lee
E: charlotte.lee@phc.ox.ac.uk
T: +44 (0)1865 289284

Word count: 3920 (excl. title page, abstract, author statement, references, and tables)

For peer review only - http://bmjopen.bmj.com/site/about/guidelines.xhtml

Abstract

Introduction: People with serious mental illness (SMI), which includes people with diagnoses of schizophrenia spectrum and bipolar disorders, face significant health inequality. This includes increased cardiovascular disease (CVD) morbidity and mortality compared with the general population. Excess weight gain and related co-morbidities are preventable risk factors for CVD in people with SMI. To improve the understanding and management of excess weight and CVD in people with SMI, we will examine the association between SMI and: (1) mean weight change; (2) cardio-metabolic risk factors for CVD; and (3) incidence of CVD and CVD fatalities. We will also (4) examine the prevalence of referral to weight management services for people with SMI compared with people without SMI.

Methods and Analysis: In this retrospective cohort study, we will link general practice records from the United Kingdom (UK) Clinical Practice Research Datalink (CPRD) Aurum database between 1998 and 2020. We will establish a cohort of patients with an SMI diagnosis matched with four controls without a SMI diagnosis on age, sex, general practice, and calendar year. We will use multivariable mixed-effects linear regression models and Cox proportional hazard models with sequential adjustment for potential confounders identified by separate directed acyclic graphs.

Ethics and Dissemination: This study has been reviewed and approved by the Independent Scientific Advisory Committee for Medicines and Healthcare products Regulatory Agency (MHRA) database research. The results will be published in a peer-reviewed journal.

Abstract word count: 234

Key words: serious mental illness, psychosis, primary care, cohort study, weight, cardiovascular

Article summary

Strengths and limitations of this study

- The key strengths of the study include the representative population contained in the CPRD, the sizeable and linked dataset, and substantial statistical power.
- The results will offer a foundation to assess the degree to which the current focus on cardiovascular prevention in people with serious mental illness is having clinical benefits.
- However, limitations include its observational nature and potential for residual confounding due to lack of information on some factors.

Introduction

Serious mental illness (SMI) that include psychotic disorders like schizophrenia spectrum and bipolar disorders are marked by hearing, seeing, or believing things that are not real.[1] People with SMI have a life expectancy that is approximately 15 to 20 years lower than the general population.[2] Approximately two-thirds of premature mortality in this group is explained by cardiovascular disease

For peer review only - http://bmjopen.bmj.com/site/about/guidelines.xhtml

For peer review only

(CVD) although suicide is also a contributing factor.[3] In fact, a 2017 meta-analysis reported people with SMI have a significantly increased risk of CVD and CVD-related mortality than the general population: hazard ratio [HR]: 1·78, 95% confidence interval [CI]: 1·60, 1·98.[4] This is startling given that CVD is the leading cause of death claiming around 17.8 million lives worldwide, and indicates an important clinical need to identify the factors underlying CVD for this group.[5,6]

It is well-established that overweight (body mass index [BMI] 25-29.9kg/m^2) and obesity (BMI ≥30 kg/m^2) are causally linked to cardio-metabolic abnormalities like insulin resistance, dyslipidaemia, and hypertension, which are major preventable risk factors for CVD. Currently, 73·6% of adults in the United States (US) and 62·3% of adults in the United Kingdom (UK) are classified as overweight or obese.[7] Yet, the prevalence of overweight and obesity has not been consistently reported for people with SMI. A 2018 report by Public Health England estimates people with SMI are three times more likely to have obesity for ages 15 to 34, and 1·6 times more likely for ages 55 to 74 compared with the general population.[8] However, other estimates from observational studies are often drawn from cross-sectional analyses of small numbers of people attending specific services within a geographical area with no comparison group.[4] Moreover, long-term data examining how weight changes over time is scarce for people with SMI.

The factors underlying excess weight gain in people with SMI are multifactorial. Psychotropic drugs (i.e., antipsychotics, antidepressants, mood stabilisers) are the mainstay treatment for the symptoms of psychosis. Evidence from clinical trials have linked these drugs to rapid weight gain but the long-term effects after a diagnosis are unclear.[9] In one 11-year follow-up study of people with schizophrenia, increased CVD mortality was reported in people treated with quetiapine (adjusted HR: 1·41, 95% CI: 1·09, 1·82) and reduced CVD mortality in people treated with clozapine (adjusted HR: 0·74, 95% CI: 0·60, 0·91).[10] However, this study is limited by unmeasured confounding of some demographic factors.[11] Accordingly, a better understanding of the long-term cardio-metabolic effects of weight gain is warranted.

Other factors that cause weight gain include unhelpful dietary patterns and physical inactivity. These behaviours are more apparent in people with SMI compared with the general population and may reflect the social disadvantage to which SMI contributes.[12] In addition, the prevalence of smoking is estimated twice that in people with SMI compared with general population,[13] and evidence shows people with schizophrenia smoke more cigarettes per day and also more intensely than the general population of smokers.[14] Other studies using polygenic risk scores for liability to schizophrenia find no association between those scores and physical health outcomes except for conditions mediated through obesity and smoking.[15] This suggests that the risks to people with SMI are modifiable.

Since obesity is modifiable, US and UK clinical guidelines recommend intervening for the prevention and management of obesity. The first-line treatment is a general practitioner (GP) referral to a community weight management programme (CWMP) to support an energy-restricted diet and increase physical activity for people with a body mass index (BMI) of 25kg/m^2 or higher.[16,17] In the general population, trials of such programmes lead to greater weight-loss than self-directed weight-loss attempts.[18] Yet, another study reported the rates of referral between 2005 and 2012 was 20·0 (95% CI: 19.3 to 20·8) per 1000 person-years, compared with weight-related advice at 30·3 (29·3 to 31·4).[19] This rate is low, and it could be that some interventions are not recorded, but other evidence

For peer review only - http://bmjopen.bmj.com/site/about/guidelines.xhtml

also point to low rates of intervention.[20] However, we have no data on whether GPs are using the annual review of people with SMI, which focuses specifically on preventative healthcare, to provide support for weight loss such as referrals to CWMPs.

We have been unable to find studies that have followed people with SMI over several years to examine how weight and cardio-metabolic profiles change. Importantly, it is not clear what drives those changes, which may include psychotropic medication and/or weight management programmes. The longest study to date was an 18-month trial of people with schizophrenia. In this study, participants randomised to olanzapine had greater increases in weight, glycaemia, and adverse lipid profile than other antipsychotics.[21] But randomised controlled trials (RCTs) are also limited to short follow-up, selected populations, and methodological issues surrounding imputation strategies used to address high dropout rates.[22][23] To that end, we do not know the extent to which weight change explains the cardio-metabolic abnormalities and higher incidence of and mortality from CVD in people with SMI.

To summarise, primary care is suitable to monitor and opportunistically offer a referral to a weight management programme for the prevention and management of obesity and its associated risks. This is true in both the general population and people with SMI who are at even higher risk for adverse physical health. However, longitudinal data outlining weight and cardio-metabolic change, the association with incidence of and mortality from CVD, and how this is currently managed for people with SMI is lacking. In this protocol, we outline our plans to address this gap in a large, retrospective, population-based study in people with SMI matched with controls without SMI in the UK from 1998 to 2020. Our objectives are:

- To examine the association between SMI and mean weight change;
- To examine the association between SMI and change in cardio-metabolic risk factors for CVD;
- To examine the association between SMI and incidence of CVD and CVD fatalities;
- To compare prevalence of offer and actual referral to weight management services in people with both SMIs and obesity (BMI ≥ 30 kg/m^2) with controls.

Methods and analysis

a. Patient and public involvement

We consulted 12 members of the public with lived experience of SMI to ensure the outcomes that matter to them are included in the protocol. Their continued involvement will inform the dissemination of our results.

b. Design

This protocol is written according to the 'REporting of studies Conducted using Observational Routinely-collected health Data' (RECORD) Statement.[24] We will use a retrospective cohort design using individual patient data from a large primary care database and a comparison cohort to examine all objectives. The planned study start date is 01/09/2021 and end date is 31/08/2024.

For peer review only - http://bmjopen.bmj.com/site/about/guidelines.xhtml

c. Data Source

We will use the Clinical Practice Research Datalink (CPRD) Aurum – a governmental, not-for-profit research service holding anonymised primary care records drawn from participating general (primary care) practices dating back to 1987 (http://www.cprd.com). To date, it encompasses routinely collected patient data records of around 13 million patients who are registered with some 700 participating general practices. Patient demographic data (i.e., year of birth, sex, and ethnicity), characteristics (i.e., height, weight, smoking status), symptoms, clinical diagnoses, consultations, referrals, prescribed medications and results of investigations are available.

d. Data Linkage

Data will be linked to the Hospital Episode Statistics (HES) and the Office for National Statistics (ONS) mortality databases. Data linkage to the Practice Level Index of Multiple Deprivation (IMD), as a proxy measure for socio-economic status (SES), will also be used when describing the baseline characteristics in the descriptive analyses and in secondary analyses.

e. Population

Source Population:

The source population will be selected from the CPRD Aurum. The source population will include all UK National Health Service (NHS) patients (male/female) registered with a participating general practice who are eligible for data linkage. We will not include up-to-standard (UTS) records because this is not yet available as a measure of data quality in the CPRD Aurum database.

Study Cohort:

From the source population, a study cohort will be selected to investigate objectives 1 to 3. We will identify and extract the records in the primary care and HES dataset for all patients with a diagnosis of SMI (hereafter *exposed*) and who are eligible for data linkage. Diagnostic Read codes, which are the standard clinical codes used in UK general practice, will be used to identify an SMI diagnosis. We will also use codes according to the International Classification of Diseases version 10 (ICD-10) to identify indications. The date of the first SMI Read code will be used as a proxy for the date of diagnosis as well as the index date, which is the earliest date that an exposed may enter the study. From the study cohort, a sub-cohort of patients will be selected to investigate objective 4. To enter the sub-cohort, the exposed need a recorded BMI ≥ 30 kg/m^2 at any time after the index date. All information will be extracted for the period 01/01/1998 to 31/10/2020 – the earliest and latest dates respectively that data are available.

Exclusion Criteria:

Patients will be excluded from the cohort(s) if they: less than one-year of active data in their participating practice before the index date; and/or who have a Read code for coronary heart disease, congestive heart failure or cardiomyopathy, and/or stroke before the index date. A flow diagram will present the selection and exclusion (with reasons where possible) of patients in the study.

For peer review only - http://bmjopen.bmj.com/site/about/guidelines.xhtml

Duration of Follow-Up:

Patients will be followed-up in the database from their index date until the earliest of the following: date of death; date of outcome of interest; date of leaving the participating practice; date of the latest download of data; or the end of the study follow-up period.

Comparison Group:

The unexposed represents the counterfactual and will include patients without an SMI Read code ever (hereafter *unexposed*) drawn from the source population. Up to four unexposed matches will be selected at baseline only if they have a qualifying weight Read code within +/- one year of the index date to the exposed in which they are matched. The matching variables include: age, sex, practice, and calendar time. All weight Read codes will be extracted.

f. Exposures

For all analyses, the exposure of interest is SMI. We will extract information on the study cohort up to end of follow-up. Extracted information includes the diagnosis date and diagnosis type, which will be grouped according to the two major SMI sub-groups as classified by the ICD-10: F20-29 non-affective psychotic disorder (i.e., schizophrenia, schizotypal and delusional disorders, psychosis not otherwise specified [NOS]) and F30-39 affective psychotic disorder (i.e., bipolar, manic, schizoaffective disorders, major depressive disorder with psychosis). It is possible patients will have Read codes in the primary care data relating to both non-affective and affective psychotic disorders. These will be classified using the most recent Read code insofar we assume that the latest diagnosis is the correct one. Based on similar studies, instances where there are Read codes relating to both diagnoses at the same date will be coded under non-affective psychotic disorder.[25]

g. Outcomes

The primary aim is objective 1 and our outcome is weight change. A preliminary search of the database indicated waist circumference was not reliably recorded so we did not include this as a measure of weight change. We will examine the extent to which weight change explains the association between SMI and: change in cardio-metabolic risk factors for CVD (objective 2) and incidence of CVD and CVD fatalities (objective 3). We will interpret the data based on the associations for all outcomes, as well as consistency and interpretable patterns. The specific outcomes for each objective are outlined in Table 1.

Table 1. Outcome measures

Objective	Outcome	Measures
Objective 1: Weight from index date to follow-up (censored date)	Weight change	Absolute mean weight change (kg)
		Percentage weight change (kg)
		Change in body mass index (kg/m^2)
Objective 2: Biomarkers of CVD risk from index date to follow-up (censored date)	Cardio-metabolic risk	Change in fasting plasma glucose (FPG)
		Change in haemoglobin A1c (HbA1c)
		Change in mean total-cholesterol
		Change in triglycerides
		Change in low-density lipoprotein (LDL)

For peer review only - http://bmjopen.bmj.com/site/about/guidelines.xhtml

		Change in blood pressure (systolic [SBP] and diastolic [DBP])
Objective 3: Total incident CVD	Incidence of CVD and CVD fatalities	Angina diagnosed by GP
		Coronary heart disease
		Congestive heart failure
		Cardiomyopathy
		Total stroke
		Total fatal CVD events
Objective 4: Weight management service referral	Proportion (%) of exposed and unexposed who are recorded as offered and actually referred to weight management service	

h. Confounding

We have identified confounding variables using directed acyclic graphs (DAGs), which aims to theoretically formalise potential confounders, colliders, and biasing relationships.[26] The DAG for each objective is described in the Supplementary Material S1. The DAG for objective 1 shows one direct path from the exposure (i.e., SMI) to the outcome (i.e., weight change), and four indirect paths: via health behaviours (i.e., alcohol use, smoking, and diet), SES status, psychotropic medication, and healthcare access and provision. Diet and physical activity are latent variables in CPRD Aurum because they are unmeasured. There are three confounding paths: via age, sex, and ethnicity.[27,28] Thus, the minimally sufficient adjustment for the total effect of SMI status on weight is to adjust for age, sex, and ethnicity.

We plan to address confounding by frequency matching the exposed with unexposed on age, as well as on sex, practice, and calendar year as described above. In addition, we will fully adjust our models by considering several covariates in the relationship between the exposure and outcome (see Table 2). All categorical variables will be analysed using the natural reference category as the reference group. Data on individual prescriptions for psychotropic and cardio-metabolic medications will be extracted from the primary care data from index date until end of follow-up. For psychotropic medications, drugs will be classified according to the British National Formulary (BNF). For objective 3, we will examine the association between SMI and CVD incidence using sequential adjustment to assess the degree to which the coefficient for the association between SMI and incidence of CVD changes. For objective 4, we will assess the presence of confounding in the association between SMI and weight loss referral by comparing estimates with and without adjustment. We will use Read codes, which have been previously developed by our team, to identify outcome and covariate records.

Table 2. Confounders

**Confounders and covariates**		**Measures**
Health Behaviours	Smoking status	Categorical (0=never; 1=ex-smoker; 2=current smoker [light: 1-9 cigarettes/day; moderate: 10-19 cigarettes/day; heavy: 20+ cigarettes/day]). This is coded in GP records. We will use the most recent recorded value of smoking status at the study start date.

For peer review only - http://bmjopen.bmj.com/site/about/guidelines.xhtml

	Alcohol use	Categorical (0=non- and ex-drinkers, 1=low, 2=moderate, 3=high, 4=dependency). This is coded in GP records. We will use the most recent recorded value of alcohol use at the study start date.
Demographics	Socioeconomic status (SES)[a]	Categorical (quintiles)
	Ethnicity[b]	Categorical (White; Asian or Asian British; Black or Black British; other ethnic group, mixed)
	Sex[b]	Binary (0=male; 1=female)
	Age at study entry, in years	Continuous
Physical and/or medical factors	Weight measured as baseline body mass index (BMI)	Categorical (normal weight [BMI 18.5-24.9 kg/m^2]; overweight [25.0-29.9]; obese [30.0-34.9]; severe obesity [35.0-35.9]; morbid obesity [40.0-44.9]
	Psychotropic medication	Categorical (antipsychotics [first- and second-generation]; antidepressants; mood stabilisers]
	Cardio-metabolic medication	Categorical (blood glucose lowering medications; antihypertensive treatments, statins)
We include the following additional variables for objectives 3 and 4 only		
Physical and/or medical factors	High cholesterol	Binary (0=no; 1=yes)
	Hypertension	Binary (0=no; 1=yes)
	Diabetes	Binary (0=no; 1=yes)
	Presence of all risk factors	Binary (0=no; 1=yes)

[a]This variable is grouped according to Practice Level Index of Multiple Deprivation (IMD). This is an indicator of material deprivation based on income; employment; health deprivation and disability; education, skills, and training; crime; barriers to housing and services; and living environment. It is categorised as quintiles 1-5 with 1 being the most deprived and 5 being the least deprived.
[b]This variable is grouped according to recommendations by the UK Office for National Statistics.[29,30]

i. Variable Library

Exposures, outcomes, and covariates of interest will be identified using medical coding (Read/SNOMED/EMIS), product coding (DM+D), and recording of patient death or transfer out of a participating practice in CPRD Aurum. The final definitions and code library has been drawn up and validated through consensus agreement amongst all investigators.

j. Sample Size Calculation

This cohort study will include all available adults with a diagnosis of SMI meaning the size of the sample ultimately depends on the number of patients in the dataset with a recorded SMI diagnosis. Hence, we present a basic sample calculation using parameter estimates obtained from previous studies and based on standard formulae. For the purpose of this calculation, we estimated a follow-up time for the exposed with a weight Read code in their record in the one-year *before* the index date. The

For peer review only - http://bmjopen.bmj.com/site/about/guidelines.xhtml

estimated median follow-up time for the exposed was 3.2 years (mean: 4.6). We assumed a 1% annual incidence rate of CVD meaning 3% of the study population would develop CVD over the ~3-years of follow-up estimated above.[31] We also assumed a relative risk (RR) of 1.3 for CVD in the exposed versus unexposed.[32] The *n* ratio was set at 0.25 (*exposed:unexposed* ratio 1:4), which achieves the best balance between data cost and statistical power. Our calculation suggested a minimum sample size of *n*=3,907 to be 80% powered to detect a RR of 1.3, alpha <u><</u>0.05%. The estimated number of patients in the CPRD Aurum is n~135,870.

k. Statistical analysis

Analyses will be conducted in Stata using an estimated approach (alpha <u><</u>0 · 05 two-tailed). We do not intend to impute missing data. The entry date into the analysis is the index date. The outcome date is the earliest of: (i) the date of death, or (ii) date of diagnosis of the outcome of interest. For objectives 1 and 2, we will use all repeated measures and run separate models for each analyses. For objective 3, we will use the first recorded diagnosis of the outcome of interest rather than recurrent events. Patients who do not have the outcome of interest will be censored at the earliest of: (i) date of death, (ii) date of leaving the practice, (iii) date of the latest download of data, or (iv) the study end date.

Descriptive summaries:

Frequency matching will be done to reduce potential confounding and to balance the baseline characteristics of the exposed and unexposed on age, sex, practice, and study year. Adequacy of matching will be confirmed by comparing means using independent *t*-tests. Descriptive statistics will describe the baseline characteristics of patients in the cohort. Categorical data will be presented as frequency and percentages, and continuous variables will be summarised using descriptive statistics (mean, standard deviation [SD], or median and interquartile range). Comparisons between the groups will be made using χ^2 tests and *t*-tests (or Wilcoxon rank-sum tests) as appropriate. Length of exposure and the most commonly prescribed class of psychotropic for each patient will be summarised. The causes of death will be categorised as CVD and all-cause mortality.

Primary analyses:

For objective 1, the primary outcome will be visually inspected by plotting trajectories of weight change using all available measurements for people with SMI versus without SMI. Then, for both objectives 1 and 2, we will use a mixed-effects linear regression model using SMI x time and psychotropic medications x time to estimate regression coefficients for mean weight change and cardio-metabolic risk factors with 95% CIs by SMI status. We will include a random effect for practice identifier to account for intra-class correlation (ICC) resulting from common practice membership. Diagnostic tests will be conducted to confirm linear regression assumptions are met including linearity of residuals, residual normality using quantile-quantile (Q-Q) plots, and residual heteroscedasticity using residual versus fitted value plots. We will add interactions (e.g., age, sex, and ethnicity) and, if significant, we will assess the strength of the associations in separate sub-groups for clarity. For objective 3, we will use Kaplan-Meier survival estimates for the exposed and unexposed groups to estimate the distribution of time from index date to total and fatal CVD risk and mortality (all-cause). The statistical equivalence of the two curves will be examined using the log-rank test. This analysis will consider survival from index date between the exposed and unexposed groups using an adjusted Cox proportional hazards regression model with follow-up time as the timescale. Restricted cubic splines

For peer review only - http://bmjopen.bmj.com/site/about/guidelines.xhtml

will be computed with five knots to visually explore non-linear associations between the exposure and the outcomes. For objective 4, we will use a Poisson regression examining person-time to estimate the proportion of patients who received weight management interventions over the study period by diagnosis and BMI category and to investigate variables associated with referral.

Sensitivity and secondary analyses:

If necessary, data on missing confounders and covariates will be addressed by adjusting for missingness as a category. Sensitivity analyses will examine the impact of running the analyses with everyone included as discussed above and confined only to those with complete data. A second sensitivity analysis will be performed excluding patients that experience the outcome within two years of the SMI Read code to avoid reversed causality from other underlying disease.

Ethics and dissemination

This study has been approved by the Independent Scientific Advisory Committee (ISAC) to the Medicines and Healthcare Products Regulatory Agency (reference: 20_000186). To guarantee the confidentiality of patient information, only the authors will have access to the data during the study. The full statistical code will be available from the authors after the publication of the results. The results of this study will be presented in scientific conferences and published in peer-reviewed journals.

Discussion

In the UK, the NHS Long Term Plan commits the health service to greater action on reducing inequalities and narrowing variation in health outcomes for marginalised and the most vulnerable groups. The plan sets out specific actions, for example, by reducing obesity in people given mental health diagnoses through physical health checks and upstream prevention. The plan also commits the NHS to support people with SMI in greater choice and control over their healthcare.

This study responds to this national agenda by leveraging the power of a large UK primary care database to provide new insight on how these actions may be achieved. Our results will estimate the long-term likelihood of weight gain in people with SMI, which could inform medication choice for both people with SMI and clinicians, particularly if there are meaningful differences between psychotropic medications (objective 1). Assessing the incidence of cardio-metabolic factors (i.e., glycaemia, adverse lipid profiles, and hypertension) relative to those without SMI will be valuable because these are key health outcomes that are being addressed (objective 2). Assessing whether the increased risk in CVD is due to changes in cardio-metabolic factors will help us understand the degree to which these are valuable therapeutic targets (objective 3). Understanding whether this group are being offered support to lose weight informs national initiatives to overcome therapeutic nihilism. It is also important to understand whether there are differences in response between people with SMI and the general population, which would inform us about whether brief interventions need to be tailored to people with SMI for more personalised care (objective 4). Ultimately, the results will offer a foundation to assess the degree to which the current focus on CVD prevention in people with SMI is having clinical benefits.

For peer review only - http://bmjopen.bmj.com/site/about/guidelines.xhtml

Since this study is observational, there are inherent strengths and limitations that are discussed. A strength of the study is its use of a representative, validated, and sizeable dataset. Furthermore, the CPRD Aurum is an appropriate data source utilising primary care records, which minimises selection, recall, and respondent bias since UK general practices tend to have good levels of accuracy in recording prescriptions and clinical diagnoses. The results will also have direct and important applications to international guidelines on patient healthcare management, as well as to British primary care physicians, mental health services, and members of the public with experience of SMI, to initiate individual action. Lastly, the longitudinal design is useful in addressing important clinical questions not usually possible within RCTs, and a matched-cohort improves efficiency since we will not need to include all controls, whilst tackling confounding.

However, since the study is descriptive, any association observed would not imply an SMI diagnosis is causative of adverse physical health outcomes. Nonetheless, we might assume the greater the magnitude of the observed association the less likely it is due to bias and more likely due to a causal process.[33] Also, diet and physical activity are also unmeasured confounders since they are unobserved in CPRD Aurum. Similarly, other unmeasured confounders like poverty may place people with SMI at increased risk of homelessness, which has consequences on healthcare access and provision. We address this potential confounding by linking to Patient Level IMD as a proxy measure of SES, but acknowledge this may not fully capture deprivation and leaves room for residual confounding. Moreover, information bias may be present since the CPRD Aurum relies on a GP recording of health outcomes. Furthermore, we can only examine primary care prescriptions. Hence, any prescribing solely within secondary care may not be included as this was not historically routinely recorded in GP records. Thus, it is also possible we will miss the first prescription(s). Physical health checks are not always performed on random samples of the population, and it is possible that regular testing is done on people with overweight and obesity. Similarly, it is also expected that some patients will have incomplete information recorded. By determining what proportion of patients do not have appropriate information recorded, we can evaluate the scale of these problems, and highlight the need for improved recording for continuity of care between healthcare professionals.

Acknowledgements

We thank the patient and public Involvement volunteers for their time and for sharing their knowledge. We also thank the patients and practices who contribute to the CPRD database. We acknowledge that HES data that will be used in this analysis will be re-used with permission from NHS Digital, who retain the copyright.

References

1. England N. *Improving Physical Healthcare for People Living with Severe Mental Illness (SMI) in Primary Care Guidance for CCGs.*; 2018. https://www.england.nhs.uk/wp-content/uploads/2018/02/improving-physical-health-care-for-smi-in-primary-care.pdf. Accessed May 4, 2021.
2. Hennekens CH, Hennekens AR, Hollar D, Casey DE. Schizophrenia and increased risks of cardiovascular disease. *Am Heart J*. 2005;150(6):1115-1121. doi:10.1016/j.ahj.2005.02.007
3. Osborn DPJ, Levy G, Nazareth I, Petersen I, Islam A, King MB. Relative risk of cardiovascular and cancer mortality in people with severe mental illness from the United Kingdom's General Practice Research Database. *Arch Gen Psychiatry*. 2007;64(2):242-249.

For peer review only - http://bmjopen.bmj.com/site/about/guidelines.xhtml

doi:10.1001/archpsyc.64.2.242
4. Correll CU, Solmi M, Veronese N, et al. Prevalence, incidence and mortality from cardiovascular disease in patients with pooled and specific severe mental illness: a large-scale meta-analysis of 3,211,768 patients and 113,383,368 controls. *World Psychiatry*. 2017;16(2):163-180. doi:10.1002/wps.20420
5. Yusuf S, Reddy S, Ôunpuu S, Anand S. Global Burden of Cardiovascular Diseases. *Circulation*. 2001;104(22):2746-2753. doi:10.1161/hc4601.099487
6. Saha S, Chant D, McGrath J. A systematic review of mortality in schizophrenia: Is the differential mortality gap worsening over time? *Arch Gen Psychiatry*. 2007;64(10):1123-1131. doi:10.1001/archpsyc.64.10.1123
7. NHS Digital. QOF 2016/17 results. https://qof.digital.nhs.uk/. Published 2019. Accessed November 14, 2019.
8. National Mental Health Intelligence Network. Severe mental illness (SMI) and physical health inequalities: briefing - GOV.UK. Public Health England. https://www.gov.uk/government/publications/severe-mental-illness-smi-physical-health-inequalities/severe-mental-illness-and-physical-health-inequalities-briefing#fn:1. Published 2018. Accessed November 14, 2019.
9. Leslie WS, Hankey CR, Lean MEJ. Review Weight gain as an adverse effect of some commonly prescribed drugs: a systematic review. *QJM*. 2007;100:395–404. doi:10.1093/qjmed/hcm044
10. Tiihonen J, Lönnqvist J, Wahlbeck K, et al. 11-year follow-up of mortality in patients with schizophrenia: a population-based cohort study (FIN11 study). *Lancet*. 2009;374(9690):620-627. doi:10.1016/S0140-6736(09)60742-X
11. De Hert M, Correll CU, Cohen D. Do antipsychotic medications reduce or increase mortality in schizophrenia? A critical appraisal of the FIN-11 study. *Schizophr Res*. 2010;117(1):68-74. doi:10.1016/j.schres.2009.12.029
12. Teasdale SB, Ward PB, Samaras K, et al. Dietary intake of people with severe mental illness: Systematic review and meta-analysis. *Br J Psychiatry*. 2019;214(5):251-259. doi:10.1192/bjp.2019.20
13. Psychiatrists RC of PRC of. *Smoking and Mental Health*. London, UK; 2013.
14. Williams J, Ziedonis D, Abanyie F, Steinberg M, Foulds J, Benowitz N. Increased nicotine and cotinine levels in smokers with schizophrenia and schizoaffective disorder is not a metabolic effect. *Schizophr Res*. 2005;79(2-3):323-335. doi:10.1016/J.SCHRES.2005.04.016
15. Kendall KM, John A, Lee SC, et al. Impact of schizophrenia genetic liability on the association between schizophrenia and physical illness: data-linkage study. *BJPsych Open*. 2020;6(6). doi:10.1192/bjo.2020.42
16. Agency for Healthcare Research and Quality. U.S. Preventive Services Task Force. Rockville, MD. https://www.ahrq.gov/cpi/about/otherwebsites/uspstf/index.html. Published 2019.
17. The National Institute for Health and Care Excellence (NICE). Obesity prevention clinical guideline [CG43]. United Kingdom. https://www.nice.org.uk/guidance/CG43. Published 2015.
18. Jolly K, Lewis A, Beach J, et al. Comparison of range of commercial or primary care led weight reduction programmes with minimal intervention control for weight loss in obesity: Lighten Up randomised controlled trial. *BMJ*. 2011;343(7832):1035. doi:10.1136/bmj.d6500
19. Booth HP, Prevost AT, Gulliford MC. Access to weight reduction interventions for overweight and obese patients in UK primary care: Population-based cohort study. *BMJ Open*. 2015;5(1):e006642. doi:10.1136/bmjopen-2014-006642
20. Humphreys K, Blodgett JC, Roberts LW. The exclusion of people with psychiatric disorders from medical research. *J Psychiatr Res*. 2015;70:28-32. doi:10.1016/j.jpsychires.2015.08.005
21. Lieberman JA, Stroup TS, McEvoy JP, et al. Effectiveness of Antipsychotic Drugs in Patients with Chronic Schizophrenia. *N Engl J Med*. 2005;353(12):1209-1223. doi:10.1056/nejmoa051688
22. Jones PB, Barnes TRE, Davies L, et al. Randomized controlled trial of the effect on quality of

For peer review only

For peer review only - http://bmjopen.bmj.com/site/about/guidelines.xhtml

life of second- vs first-generation antipsychotic drugs in schizophrenia: Cost Utility of the Latest Antipsychotic Drugs in Schizophrenia Study (CUtLASS 1). *Arch Gen Psychiatry*. 2006;63(10):1079-1087. doi:10.1001/archpsyc.63.10.1079

23. Álvarez-Jiménez M, González-Blanch C, Crespo-Facorro B, et al. Antipsychotic-induced weight gain in chronic and first-episode psychotic disorders: A systematic critical reappraisal. *CNS Drugs*. 2008;22(7):547-562. doi:10.2165/00023210-200822070-00002
24. Benchimol E, Smeeth L, Guttmann A, et al. The REporting of studies Conducted using Observational Routinely-collected health Data (RECORD) statement. *PLoS Med*. 2015;12(10). doi:10.1371/JOURNAL.PMED.1001885
25. Hope H, Parisi R, Ashcroft DM, et al. Fertility trends of women with serious mental illness in the United Kingdom 1992–2017: A primary care cohort study using the clinical practice research datalink. *J Affect Disord*. 2020;269:141-147. doi:10.1016/j.jad.2020.03.037
26. Shrier I, Platt RW. Reducing bias through directed acyclic graphs. *BMC Med Res Methodol*. 2008;8:70. doi:10.1186/1471-2288-8-70
27. Merikangas K, He J, Burstein M, et al. Lifetime prevalence of mental disorders in U.S. adolescents: results from the National Comorbidity Survey Replication--Adolescent Supplement (NCS-A). *J Am Acad Child Adolesc Psychiatry*. 2010;49(10):980-989. doi:10.1016/J.JAAC.2010.05.017
28. Schwartz RC. Racial disparities in psychotic disorder diagnosis: A review of empirical literature. *World J Psychiatry*. 2014;4(4):133. doi:10.5498/wjp.v4.i4.133
29. Government Statistical Service. Ethnicity harmonised standard. https://gss.civilservice.gov.uk/policy-store/ethnicity/. Published 2019. Accessed August 13, 2021.
30. Government Statistical Service. Sex and gender harmonisation guidance. https://gss.civilservice.gov.uk/policy-store/sex-and-gender/. Published 2019. Accessed August 13, 2021.
31. Fan Z, Wu Y, Shen J, Ji T, Zhan R. Schizophrenia and the risk of cardiovascular diseases: A meta-analysis of thirteen cohort studies. *J Psychiatr Res*. 2013;47(11):1549-1556. doi:10.1016/j.jpsychires.2013.07.011
32. Cunningham R, Poppe K, Peterson D, Every-Palmer S, Soosay I, Jackson R. Prediction of cardiovascular disease risk among people with severe mental illness: A cohort study. *PLoS One*. 2019;14(9). doi:10.1371/journal.pone.0221521
33. Grimes DA, Schulz KF. Bias and causal associations in observational research. *Lancet*. 2002;359(9302):248-252. doi:10.1016/S0140-6736(02)07451-2

For peer review only

Footnotes

Contributions

CL, FW, PA, and CPS conceived the study idea. CL, FW, MS, MG, CB, PA, and CP contributed to the development of the study design and protocol. CL drafted the manuscript. All authors approved the final submitted version.

Funding

This research received no specific grant from any funding agency in the public, commercial or not-for-profit sectors. CL is funded by the Engineering and Physical Sciences Research Council (EPSRC; EP/R513295/1) and the National Institute for Health Research (NIHR) Oxford Biomedical Research Centre (BRC; IS-BRC-1215-20008). MS is supported by the NIHR Oxford BRC. CB is supported by the NIHR Oxford BRC. PA is funded by the NIHR BRC, the NIHR ARC (200172), and is an NIHR senior

For peer review only - http://bmjopen.bmj.com/site/about/guidelines.xhtml

investigator (NF-SI-0617-10064). CPS is funded through the NIHR ARC. FW is funded by a Wellcome Trust Clinical Doctoral Fellowship (102176/B/13/Z).

Disclaimer

The views expressed are those of the author(s) and not necessarily those of the NIHR or the Department of Health and Social Care.

Competing Interests

PA has conducted a trial funded by Cambridge Weight Plan and has spoken at a seminar at the Royal College of General Practitioners (RCGP) conference that was sponsored by Novo Nordisk. None of these activates led to personal payments to him. All other authors declare no conflict of interests.

Patient consent for publication

Not required.

Data statement

The data was made available by the CPRD obtained under licence from the UK MRHA. Data is available to researchers who are approved by the Independent Scientific Advisory Committee.

For peer review only

Weight change, cardio-metabolic risk factors, and cardiovascular incidence in people with serious mental illness: protocol of a population-based cohort study in the UK from 1997 – 2020

Supplementary Material

Supplementary material S1. Direct acyclic graphs (DAGs) for the association between SMI and each outcome.

In the following figures, the green box (i.e., SMI status) is the exposure variable. The blue box 'I' is the outcome variable. The grey boxes 'diet' and 'physical activity' are latent variables because they are unobserved in the CPRD Aurum dataset. Causal paths are indicated via green arrows; biasing paths in red; and paths with no influence on the outcome in black.

Figure 1. DAG for the association between SMI and mean weight change (objective 1).

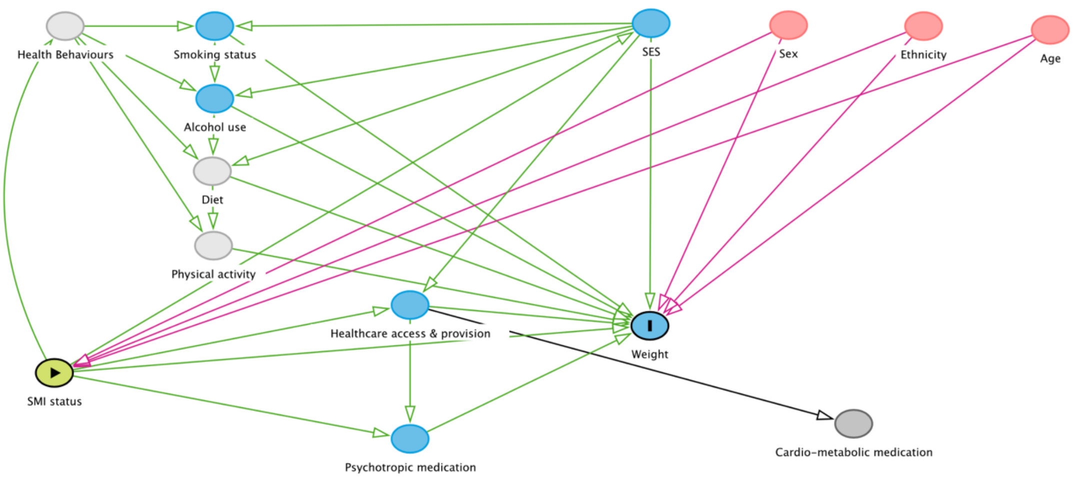

This DAG shows **five** paths between the exposure (i.e., SMI status) and outcome (i.e., weight): the (1) direct path from SMI status to weight; the indirect paths via (2) health behaviours (i.e., smoking status, alcohol use, diet, and physical activity), via (3) socio-economic status (SES), via (4) psychotropic medications, and via (5) healthcare access and provision. All variables on the indirect paths are mediating. There are three confounding paths: the path via age, sex, and ethnicity. Thus, the minimally sufficient adjustment to get the total effect of the exposure on the outcome is to adjust for age, sex, and ethnicity. The direct effect cannot be estimated by covariate adjustment.

Figure 2. DAG for the association between SMI and biomarkers for CVD risk (objective 2).

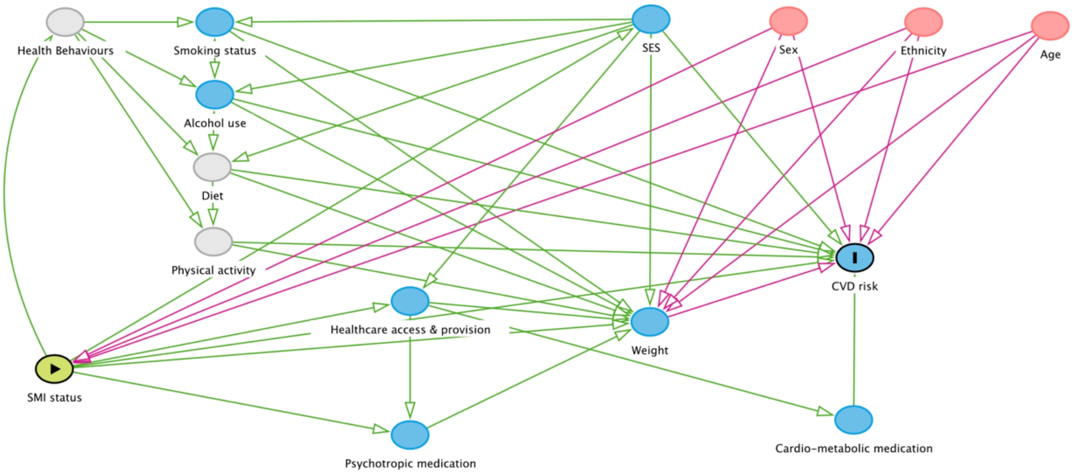

This DAG shows **seven** paths between the exposure (i.e., SMI status) and outcome (i.e., CVD risk): the (1) direct path from SMI status to CVD risk; the indirect paths via (2) health behaviours (i.e., smoking status, alcohol use, diet, physical activity), via (3) SES, via (4) psychotropic medications, via (5) healthcare access and provision, via (6) cardio-metabolic medication, and via (7) weight. There are three confounding paths: the path via age, sex, and ethnicity. Thus, the minimally sufficient adjustment to get the total effect of the exposure on the outcome is to adjust for age, sex, and ethnicity. The direct effect cannot be estimated by covariate adjustment.

For peer review only

Figure 3. DAG for the association between SMI and CVD incidence (objective 3).

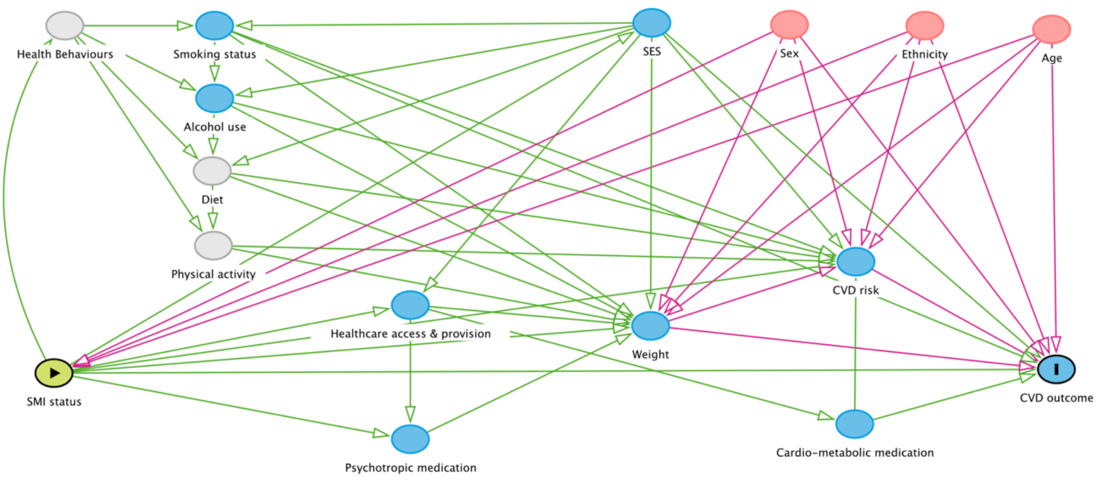

This DAG shows **eight** paths between the exposure (i.e., SMI status) and outcome (i.e., CVD risk): the (1) direct path from SMI status to CVD risk; the indirect paths via (2) health behaviours (i.e., smoking status, alcohol use, diet, and physical activity), via (3) SES, via (4) psychotropic medications, via (5) healthcare access and provision, via (6) cardio-metabolic medication, via (7) weight, and via (8) CVD risk. The minimally sufficient adjustment to get the total effect of the exposure on the outcome is to adjust for age, sex, and ethnicity. The minimally sufficient adjustment for estimating the direct effect of the exposure on the outcome is to adjust for: age, sex, ethnicity, SES, smoking, weight, cardio-metabolic medication, and CVD risk.

For peer review only

Figure 4. DAG to compare the prevalence of offer and actual referral to weight management services in people with both SMI and obesity with controls (objective 4).

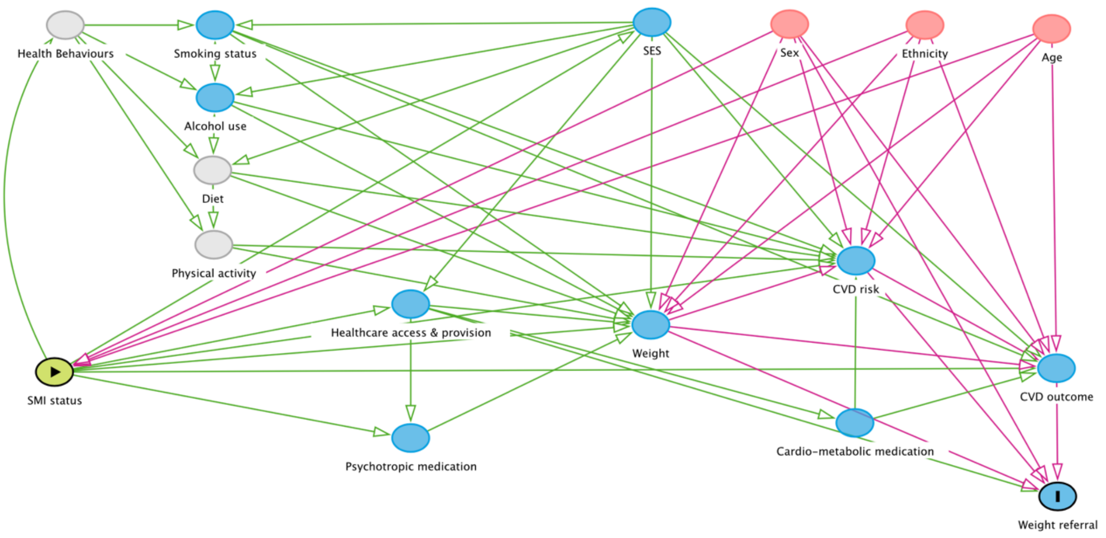

This model does not change the adjustment set.

For peer review only

The RECORD statement – checklist of items, extended from the STROBE statement, that should be reported in observational studies using routinely collected health data.

	Item No.	STROBE items	Location in manuscript where items are reported	RECORD items	Location in manuscript where items are reported
**Title and abstract**					
	1	(a) Indicate the study's design with a commonly used term in the title or the abstract (b) Provide in the abstract an informative and balanced summary of what was done and what was found	(a): Page 1 – Title; Page 2 – Abstract<br><br>(b): Not applicable because this manuscript reports a protocol	RECORD 1.1: The type of data used should be specified in the title or abstract. When possible, the name of the databases used should be included.<br><br>RECORD 1.2: If applicable, the geographic region and timeframe within which the study took place should be reported in the title or abstract.<br><br>RECORD 1.3: If linkage between databases was conducted for the study, this should be clearly stated in the title or abstract.	(1.1): Page 2 - Abstract<br><br>(1.2): Page 2 - Abstract<br><br>(1.3): Page 2 - Abstract
**Introduction**					
Background rationale	2	Explain the scientific background and rationale for the investigation being reported	Page 3 – Background		
Objectives	3	State specific objectives, including any prespecified hypotheses	Page 4 – Background		
**Methods**					
Study Design	4	Present key elements of study design early in the paper	Methods and analysis:<br>• Page 4 - Design		
Setting	5	Describe the setting, locations, and relevant dates, including periods of recruitment, exposure, follow-up, and data collection	Methods and analysis:<br>• Settings, locations, data collection: Page 4 - Data Source<br>• Periods of recruitment: Page 5 - Study Cohort<br>• Exposure: Page 6 - Exposures<br>• Follow-up: Page 5 –		

For peer review only

			Duration of Follow-Up		
Participants	6	*(a) Cohort study* - Give the eligibility criteria, and the sources and methods of selection of participants. Describe methods of follow-up<br>*Case-control study* - Give the eligibility criteria, and the sources and methods of case ascertainment and control selection. Give the rationale for the choice of cases and controls<br>*Cross-sectional study* - Give the eligibility criteria, and the sources and methods of selection of participants<br><br>*(b) Cohort study* - For matched studies, give matching criteria and number of exposed and unexposed<br>*Case-control study* - For matched studies, give matching criteria and the number of controls per case	(a): Page 5 – Study Cohort<br><br>(b): Page 5 – Comparison Group	RECORD 6.1: The methods of study population selection (such as codes or algorithms used to identify subjects) should be listed in detail. If this is not possible, an explanation should be provided.<br><br>RECORD 6.2: Any validation studies of the codes or algorithms used to select the population should be referenced. If validation was conducted for this study and not published elsewhere, detailed methods and results should be provided.<br><br>RECORD 6.3: If the study involved linkage of databases, consider use of a flow diagram or other graphical display to demonstrate the data linkage process, including the number of individuals with linked data at each stage.	(6.1): Page 5 – Study Cohort<br><br>(6.2): Not done because this manuscript reports a protocol. We will do this for any output papers.<br><br>(6.3): Considered but not thought necessary.
Variables	7	Clearly define all outcomes, exposures, predictors, potential confounders, and effect modifiers. Give diagnostic criteria, if applicable.	Methods and analysis:<br>• Exposures: Page 6 – Exposures<br>• Potential confounders and effect modifiers – Page 7 – Confounding	RECORD 7.1: A complete list of codes and algorithms used to classify exposures, outcomes, confounders, and effect modifiers should be provided. If these cannot be reported, an explanation should be provided.	(7.1): Not provided because this manuscript reports a protocol. We will provide this for any output papers.
Data sources/ measurement	8	For each variable of interest, give sources of data and details of methods of assessment (measurement). Describe comparability of assessment methods if there is more than one group	Not considered		
Bias	9	Describe any efforts to address potential sources of bias	Page 7 - Confounding		
Study size	10	Explain how the study size was arrived at	Page 8 – Sample size calculation		
Quantitative variables	11	Explain how quantitative variables were handled in the analyses. If	Page 9 – Primary analyses		

For peer review only

		applicable, describe which groupings were chosen, and why			
Statistical methods	12	(a) Describe all statistical methods, including those used to control for confounding<br>(b) Describe any methods used to examine subgroups and interactions<br>(c) Explain how missing data were addressed<br>(d) *Cohort study* - If applicable, explain how loss to follow-up was addressed<br>*Case-control study* - If applicable, explain how matching of cases and controls was addressed<br>*Cross-sectional study* - If applicable, describe analytical methods taking account of sampling strategy<br>(e) Describe any sensitivity analyses	(a): Page 9 – Statistical methods (primary analyses)<br><br>(b): Page 9 – Statistical methods (primary analyses)<br><br>(b): Page 9 – Statistical methods (sensitivity and secondary analyses)		
Data access and cleaning methods		..		RECORD 12.1: Authors should describe the extent to which the investigators had access to the database population used to create the study population.<br><br>RECORD 12.2: Authors should provide information on the data cleaning methods used in the study.	(12.1): Not considered in the manuscript. But bull access will available.<br><br>(12.2): Not yet performed.
Linkage		..		RECORD 12.3: State whether the study included person-level, institutional-level, or other data linkage across two or more databases. The methods of linkage and methods of linkage quality evaluation should be provided.	(12.3): Page 5 – Data Linkage
**Results**					
Participants	13	(a) Report the numbers of individuals at each stage of the study (*e.g.*, numbers potentially eligible, examined for eligibility, confirmed eligible, included in the study, completing follow-up, and analysed)<br>(b) Give reasons for non-participation at each stage.<br>(c) Consider use of a flow diagram	(a-c): Not applicable	RECORD 13.1: Describe in detail the selection of the persons included in the study (*i.e.*, study population selection) including filtering based on data quality, data availability and linkage. The selection of included persons can be described in the text and/or by means of the study flow diagram.	(13.1): Not applicable
Descriptive data	14	(a) Give characteristics of study participants (*e.g.*, demographic, clinical, social) and information on exposures and potential confounders	(a-c): Not applicable		

For peer review only - http://bmjopen.bmj.com/site/about/guidelines.xhtml

		(b) Indicate the number of participants with missing data for each variable of interest<br>(c) *Cohort study* - summarise follow-up time (*e.g.*, average and total amount)			
Outcome data	15	*Cohort study* - Report numbers of outcome events or summary measures over time<br>*Case-control study* - Report numbers in each exposure category, or summary measures of exposure<br>*Cross-sectional study* - Report numbers of outcome events or summary measures	Not applicable		
Main results	16	(a) Give unadjusted estimates and, if applicable, confounder-adjusted estimates and their precision (e.g., 95% confidence interval). Make clear which confounders were adjusted for and why they were included<br>(b) Report category boundaries when continuous variables were categorized<br>(c) If relevant, consider translating estimates of relative risk into absolute risk for a meaningful time period	(a-c): Not applicable		
Other analyses	17	Report other analyses done—e.g., analyses of subgroups and interactions, and sensitivity analyses	Not applicable		
Key results	18	Summarise key results with reference to study objectives	Not applicable		
Limitations	19	Discuss limitations of the study, taking into account sources of potential bias or imprecision. Discuss both direction and magnitude of any potential bias	Page 9-10 - Discussion	RECORD 19.1: Discuss the implications of using data that were not created or collected to answer the specific research question(s). Include discussion of misclassification bias, unmeasured confounding, missing data, and changing eligibility over time, as they pertain to the study being reported.	Not applicable
Interpretation	20	Give a cautious overall interpretation of results considering objectives, limitations, multiplicity of analyses, results from similar studies, and other relevant evidence	Not applicable		
Generalisability	21	Discuss the generalisability (external validity) of the study results	Page 9-10 - Discussion		
**Other Information**					

For peer review only

For peer review only - http://bmjopen.bmj.com/site/about/guidelines.xhtml

Funding	22	Give the source of funding and the role of the funders for the present study and, if applicable, for the original study on which the present article is based	Page 10 – Acknowledgements (Funding)		
Accessibility of protocol, raw data, and programming code		..		RECORD 22.1: Authors should provide information on how to access any supplemental information such as the study protocol, raw data, or programming code.	Page 10 – Acknowledgements (Ethics and dissemination)

*Reference: Benchimol EI, Smeeth L, Guttmann A, Harron K, Moher D, Petersen I, Sørensen HT, von Elm E, Langan SM, the RECORD Working Committee. The REporting of studies Conducted using Observational Routinely-collected health Data (RECORD) Statement. *PLoS Medicine* 2015; in press.

*Checklist is protected under Creative Commons Attribution (CC BY) license.

For peer review only

BMJ Open

Weight change, cardio-metabolic risk factors, and cardiovascular incidence in people with serious mental illness: protocol of a population-based cohort study in the UK from 1998 to 2020

Journal:	*BMJ Open*
Manuscript ID	bmjopen-2021-053427.R2
Article Type:	Protocol
Date Submitted by the Author:	06-Oct-2021

Complete List of Authors:	Lee, Charlotte; University of Oxford, Nuffield Department of Primary Care Health Sciences; NIHR Oxford Biomedical Research Centre, Oxford University Hospitals NHS Foundation Trust<br>Waite, Felicity; University of Oxford, Department of Psychiatry; Oxford Health NHS Foundation Trust<br>Smith, Margaret; University of Oxford, Nuffield Department of Primary Care Health Sciences; NIHR Oxford Biomedical Research Centre<br>Gao, Min; University of Oxford, Nuffield Department of Primary Care Health Sciences; Peking University Health Science Centre, School of Public Health<br>Bankhead, Clare; University of Oxford, Nuffield Department of Primary Care Health Sciences; NIHR Oxford Biomedical Research Centre<br>Aveyard, Paul; University of Oxford, Primary Care Health Sciences; NIHR Oxford Biomedical Research Centre<br>Piernas, Carmen; University of Oxford, Nuffield Department of Primary Care Health Sciences

**Primary Subject Heading**:	Epidemiology
Secondary Subject Heading:	Mental health
Keywords:	Schizophrenia & psychotic disorders < PSYCHIATRY, PRIMARY CARE, EPIDEMIOLOGY

SCHOLARONE™
Manuscripts

I, the Submitting Author has the right to grant and does grant on behalf of all authors of the Work (as defined in the below author licence), an exclusive licence and/or a non-exclusive licence for contributions from authors who are: i) UK Crown employees; ii) where BMJ has agreed a CC-BY licence shall apply, and/or iii) in accordance with the terms applicable for US Federal Government officers or employees acting as part of their official duties; on a worldwide, perpetual, irrevocable, royalty-free basis to BMJ Publishing Group Ltd ("BMJ") its licensees and where the relevant Journal is co-owned by BMJ to the co-owners of the Journal, to publish the Work in this journal and any other BMJ products and to exploit all rights, as set out in our licence.

The Submitting Author accepts and understands that any supply made under these terms is made by BMJ to the Submitting Author unless you are acting as an employee on behalf of your employer or a postgraduate student of an affiliated institution which is paying any applicable article publishing charge ("APC") for Open Access articles. Where the Submitting Author wishes to make the Work available on an Open Access basis (and intends to pay the relevant APC), the terms of reuse of such Open Access shall be governed by a Creative Commons licence – details of these licences and which Creative Commons licence will apply to this Work are set out in our licence referred to above.

Other than as permitted in any relevant BMJ Author's Self Archiving Policies, I confirm this Work has not been accepted for publication elsewhere, is not being considered for publication elsewhere and does not duplicate material already published. I confirm all authors consent to publication of this Work and authorise the granting of this licence.

For peer review only

TITLE PAGE

Weight change, cardio-metabolic risk factors, and cardiovascular incidence in people with serious mental illness: protocol of a population-based cohort study in the UK from 1998 to 2020

Authors and Affiliations:

Charlotte Lee[a,b], Felicity Waite[c,d], Margaret Smith[a,b], Min Gao[a,e], Clare Bankhead[a,b], Paul Aveyard[a,b*], Carmen Piernas[a*]

a. Nuffield Department of Primary Care Health Sciences, University of Oxford, Oxford, OX2 6GG. United Kingdom.

b. NIHR Oxford Biomedical Research Centre, Oxford University Hospitals NHS Foundation Trust.

c. Department of Psychiatry, University of Oxford, Warneford Hospital, Warneford Lane, Headington, Oxford, OX3 7JX. United Kingdom.

d. Oxford Health NHS Foundation Trust, Warneford Lane, Headington, Oxford, OX3 7JX. United Kingdom.

e. School of Public Health, Peking University Health Science Centre, Beijing, China.

*These authors equally contributed to this work.

Corresponding Author:

Charlotte Lee
E: charlotte.lee@phc.ox.ac.uk
T: +44 (0)1865 289284

Word count: 3920 (excl. title page, abstract, author statement, references, and tables)

For peer review only

For peer review only - http://bmjopen.bmj.com/site/about/guidelines.xhtml

Abstract

Introduction: People with serious mental illness (SMI), which includes people with diagnoses of schizophrenia spectrum and bipolar disorders, face significant health inequality. This includes a life expectancy reduced by 15 to 20 years mostly due to premature cardiovascular disease (CVD) compared with the general population. Excess weight gain and related co-morbidities are preventable risk factors for CVD. To improve the understanding and management of CVD in people with SMI, we will examine the association between SMI and: (1) weight change; (2) cardio-metabolic risk factors for CVD; and (3) incidence of and mortality from CVD. We will also (4) examine the incidence of referral to weight management services for people with SMI compared with people without SMI.

Methods and Analysis: In this retrospective cohort study, we will link general practice records from the United Kingdom (UK) Clinical Practice Research Datalink (CPRD) Aurum database. We will establish a cohort of patients diagnosed with SMI between 1998 and 2020 who are matched with up to four controls on age, sex, general practice, and calendar year. We will use multivariable mixed-effects linear regression models and Cox proportional hazard models with sequential adjustment for potential confounders identified by separate directed acyclic graphs.

Ethics and Dissemination: This study has been reviewed and approved by the Independent Scientific Advisory Committee for Medicines and Healthcare products Regulatory Agency (MHRA) database research. The results will be published in a peer-reviewed journal.

Abstract word count: 232

Key words: serious mental illness, psychosis, primary care, cohort, weight, cardiovascular

Article summary

Strengths and limitations of this study

- The key strengths of the study include the representative population contained in the CPRD Aurum, the sizeable and linked dataset, and substantial statistical power.
- However, limitations include its observational nature and potential for residual confounding due to lack of information on some factors.
- There is also potential for selection bias since physical health checks are not always performed on random population samples.

Introduction

Serious mental illness (SMI) includes psychotic disorders like schizophrenia spectrum and bipolar disorders, which are marked by hearing, seeing, or believing things that are not real.[1] People with SMI have a life expectancy that is approximately 15 to 20 years lower than the general population.[2] Two-thirds of premature mortality in this group is explained by preventable cardiovascular disease (CVD).[3] A 2017 meta-analysis reported people with SMI have a significantly increased incidence of and

For peer review only - http://bmjopen.bmj.com/site/about/guidelines.xhtml

mortality from than the general population: hazard ratio [HR]: 1·78, 95% confidence interval [CI]: 1·60, 1·98.[4] This indicates an important clinical need to identify the factors underlying the higher incidence of CVD for this group and routes for prevention and treatment.[5,6]

Overweight (body mass index [BMI] 25-29.9kg/m^2) and obesity (BMI ≥30 kg/m^2) are causally linked to cardio-metabolic abnormalities like insulin resistance, dyslipidaemia, and hypertension, which are major preventable risk factors for CVD. Latest figures show that 64% of adults in the United Kingdom (UK) are classified as overweight or obese.[7] Overweight and obesity has not been consistently reported for people with SMI. A 2018 report based on a representative sample of English general practice records found that people with SMI were three times more likely to have obesity between the ages 15 to 34, and 1·6 times more likely for ages 55 to 74 compared with the general population.[8] However, these estimates were limited by high rates on non-completion of BMI in general practice. Moreover, other estimates are often drawn from cross-sectional analyses and often include small numbers of people attending specific services within a geographical area with no comparison group.[4]

The factors underlying excess weight gain in people with SMI are multifactorial. Psychotropic drugs (i.e., antipsychotics, antidepressants, mood stabilisers) are the mainstay treatment for the symptoms of psychosis and affect weight.[9] Evidence from clinical trials have linked these drugs to rapid weight gain, but the long-term effects after a diagnosis are unclear.[9] In one 11-year follow-up study of people with schizophrenia, increased CVD mortality was reported in people treated with quetiapine (adjusted HR: 1·41, 95% CI: 1·09, 1·82) and reduced CVD mortality in people treated with clozapine (adjusted HR: 0·74, 95% CI: 0·60, 0·91).[10] However, these estimates may result from unmeasured confounding by socioeconomic and other demographic factors.[11] Accordingly, a better understanding of the long-term impact of medication on weight change, cardio-metabolic risk factors, and incidence of and mortality from CVD is warranted.

Other factors that cause weight gain include unhelpful dietary patterns and physical inactivity. These behaviours are more common in people with SMI compared with the general population and may reflect the social disadvantage to which SMI contributes.[12] In addition, the prevalence of smoking is estimated to be twice that in people with SMI compared with general population,[13] and evidence shows people with schizophrenia smoke more cigarettes per day and more intensely than the general population of smokers.[14] Other studies using polygenic risk scores for liability to schizophrenia have found no association between those scores and physical health outcomes except for conditions mediated through obesity and smoking.[15] This suggests that the excess risk to people with SMI are modifiable.

Since obesity is modifiable, US and UK clinical guidelines recommend intervening to prevent and manage obesity. In the UK, the first-line intervention is general practitioner (GP) advice or a referral to a weight management programme to support an energy-restricted diet and increase physical activity for people with a BMI >25kg/m^2.[16,17] In the general population, trials of such programmes lead to greater weight-loss than self-directed attempts.[18] Another study in the general population reported the rates of referral between 2005 and 2012 was 20·0 (95% CI: 19.3 to 20·8) per 1000 person-years, compared with weight-related advice at 30·3 (29·3 to 31·4).[19] This rate is low, and although these interventions may not be recorded, other evidence also point to low rates of intervention.[20] However, we have no data on whether GPs are using the annual review of people with SMI, which focuses

For peer review only - http://bmjopen.bmj.com/site/about/guidelines.xhtml

specifically on preventative healthcare, to provide support for weight management such as referrals to weight management programme.

We have been unable to find studies that have followed people with SMI over several years to examine how weight and cardio-metabolic profiles change. It is possible these relate to ongoing psychotropic and/or weight management programmes. The longest study to date was an 18-month trial of people with schizophrenia. In this study, participants randomised to olanzapine had greater increases in weight, glycaemia, and adverse lipid profile than other antipsychotics.[21] Randomised controlled trials (RCTs) are often limited to short follow-up of selected populations, and there are methodological issues surrounding imputation strategies used to address high dropout rates.[22,23] Thus, it remains unclear how much weight and cardio-metabolic profiles change over the longer-term, and how much of the excess incidence and mortality from CVD is explained by those changes in people with SMI.

In this protocol, we outline our plans to address this gap in a large, retrospective, population-based study in people with SMI matched with controls without SMI in the UK from 1998 to 2020. Our objectives are to:

- Examine the association between SMI and weight change;
- Examine the association between SMI and change in cardio-metabolic risk factors for CVD;
- Examine the association between SMI and incidence of and mortality from CVD;
- Compare incidence of advice and offer and actual referral to weight management services in people with both SMIs and BMI ≥ 25 kg/m^2 with controls.

Methods and analysis

a. Patient and public involvement

We consulted 12 members of the public with lived experience of SMI to ensure the outcomes that matter to them are included in the protocol. Their continued involvement will inform the dissemination of our results.

b. Design

This protocol is written according to the 'REporting of studies Conducted using Observational Routinely-collected health Data' (RECORD) Statement.[24] We will use a retrospective cohort using individual patient data from a large primary care database to examine all objectives. The planned study start date is 01/09/2021 and end date is 31/08/2024.

c. Data Source

We will use the Clinical Practice Research Datalink (CPRD) Aurum – a governmental, not-for-profit research service holding anonymised primary care records drawn from participating general (primary care) practices dating back to 1987 (http://www.cprd.com). To date, it encompasses routinely collected patient data records of around 16 million patients who are currently registered with some 700 participating general practices. Patient demographic data (i.e., year of birth, sex, and ethnicity),

For peer review only - http://bmjopen.bmj.com/site/about/guidelines.xhtml

characteristics (i.e., height, weight, smoking status), symptoms, clinical diagnoses, consultations, referrals, prescribed medications and results of investigations are available.

d. Data Linkage

Data will be linked to the Hospital Episode Statistics (HES) and the Office for National Statistics (ONS) mortality databases. Data linkage to the practice level Index of Multiple Deprivation (IMD), as a proxy measure for socio-economic status (SES), will also be used when describing the baseline characteristics in the descriptive analyses and in secondary analyses.

e. Population

Source Population:

The source population will be selected from the CPRD Aurum. The source population will include all UK National Health Service (NHS) patients (male/female) registered with a participating general practice who are eligible for data linkage. We will not include up-to-standard (UTS) records because this is not yet available as a measure of data quality in the CPRD Aurum database.

Study Cohort:

We will select a study cohort from the CPRD Aurum to investigate objectives 1 to 3. We will identify and extract the records in the primary care and HES dataset for all patients with a diagnosis of SMI (hereafter *exposed*) and who are eligible for data linkage. Diagnostic Read codes, which are the standard clinical codes used in UK general practice, will be used to identify an SMI diagnosis. We will also use codes according to the International Classification of Diseases version 10 (ICD-10) from the HES data. The date of the first SMI Read code will be used as the index date, which is the earliest date that an exposed patient may enter the study. From the study cohort, a sub-cohort of patients will be selected to investigate objective 4. To enter the sub-cohort, the exposed patient needs a recorded BMI ≥ 25 kg/m^2 at any time after the index date. All information will be extracted for the period 01/01/1998 to 31/10/2020 – the earliest and latest dates respectively that data are available.

Exclusion Criteria:

Patients will be excluded from the cohort(s) if they: less than one-year of active data in their participating practice before the index date; and/or who have a Read code for coronary heart disease, congestive heart failure or cardiomyopathy, and/or stroke before the index date. A flow diagram will present the selection and exclusion (with reasons where possible) of patients in the study.

Duration of Follow-Up:

Patients will be followed-up in the database from their index date until the earliest of the following: date of death; date of outcome of interest; date of leaving the participating practice; date of the latest download of data; or the end of the study follow-up period (i.e., up to 22 years).

Comparison Group:

The unexposed represents the counterfactual and will include patients without an SMI Read code ever (hereafter *unexposed*) drawn from the source population. Up to four unexposed matches will be selected at baseline only if they have a qualifying weight Read code within +/- one year of the index

For peer review only - http://bmjopen.bmj.com/site/about/guidelines.xhtml

date to the exposed in which they are matched. The matching variables include: age, sex, practice, and calendar time. All weight Read codes will be extracted.

f. Exposures

For all analyses, the exposure of interest is SMI. We will extract information on the study cohort up to censoring. Extracted information includes the diagnosis date and type, which will be grouped according to the two major SMI sub-groups as classified by the ICD-10: F20-29 non-affective psychotic disorder (i.e., schizophrenia, schizotypal and delusional disorders, psychosis not otherwise specified [NOS]) and F30-39 affective psychotic disorder (i.e., bipolar, manic, schizoaffective disorders, major depressive disorder with psychosis). Patients may have Read codes in the primary care data relating to both non-affective and affective psychotic disorders. These will be classified using the most recent Read code because we will assume that the latest diagnosis is the correct one. For example, it is common for diagnoses to be clarified over time from first episode psychosis to schizophrenia. Based on similar studies, instances where there are Read codes relating to both diagnoses at the same date will be coded under non-affective psychotic disorder.[25] Data on individual prescriptions for psychotropic medication will be extracted from the primary care data from index date until censored.

g. Outcomes

The primary aim of objective 1 is to assess the impact of a SMI diagnosis on weight change. A pilot search of the database indicated waist circumference was not reliably recorded so we did not include this as a measure of adiposity. Our outcome for objective 2 are change in cardio-metabolic risk factors for CVD, which include change in systolic and diastolic blood pressure, change in total cholesterol/HDL ratio, and change in glycaemia indicated by HbA1c or fasting glucose. Our outcome for objective 3 is incidence of and mortality from CVD. Our objective for 4 is coded weight management advice and referral to weight management services. We will interpret the data based on the associations for all outcomes, as well as consistency and interpretable patterns. The specific outcomes for each objective are outlined in Table 1.

Table 1. Outcome measures

Objective	Outcome	Measures
Objective 1: Examine the association between SMI and weight change	Weight change	Absolute mean weight change (kg)
		Percentage weight change (kg)
		Change in body mass index (kg/m^2)
Objective 2: Examine the association between SMI and changes cardio-metabolic risk factors for CVD	Cardio-metabolic risk	Change in fasting blood glucose (FBG)
		Change in haemoglobin A1c (HbA1c)
		Change in mean total-cholesterol
		Change in triglycerides
		Change in low-density lipoprotein (LDL)
		Change in blood pressure (systolic [SBP] and diastolic [DBP])
Objective 3: Total incident and fatal CVD	Incidence of CVD and CVD fatalities	Angina diagnosed by GP
		Acute coronary syndrome
		Coronary heart disease
		Congestive heart failure

For peer review only - http://bmjopen.bmj.com/site/about/guidelines.xhtml

		Cardiomyopathy
		Total stroke
		Total fatal CVD events
Objective 4: Weight management advice and service referral	Proportion (%) of exposed and unexposed who are recorded as offered advice, and offered then actually referred to weight management service	

h. Confounding

We have identified confounding variables using directed acyclic graphs (DAGs), which aims to theoretically formalise potential confounders, colliders, and biasing relationships.[26] The DAG for each objective is described in the Supplementary Material S1. The DAG for objective 1 shows one direct path from the exposure (i.e., SMI) to the outcome (i.e., weight change), and four indirect paths: via health behaviours (i.e., alcohol use, smoking, and diet), SES status, psychotropic medication, and healthcare access and provision. Diet and physical activity are latent variables in CPRD Aurum because they are unmeasured. There are three confounding paths: via age, sex, and ethnicity.[27,28] Thus, the minimally sufficient adjustment for the total effect of SMI status on weight is to adjust for age, sex, and ethnicity.

Frequency matching will ensure our ability to adjust for confounding variables that include age, sex, and calendar year, and matching on practice will remove practice-specific variables. In addition, we will fully adjust our models by considering several covariates in the relationship between the exposure and outcome (see Table 2). All categorical variables will be analysed using the natural reference category as the reference group. Data on individual prescriptions for psychotropic and cardio-metabolic medications will be extracted from the primary care data from index date until censoring. For psychotropic medications, drugs will be classified according to the British National Formulary (BNF). For objective 2 and 3, we will assess the impact of weight gain on change in cardio-metabolic risk factors and incidence of CVD using mediation analysis to allow us to estimate the direct (unmediated) and indirect (mediated) effects of SMI on CVD risk and incidence. To do this, we will fit two regression models: one regressing the outcome on the exposure, adjusting for confounders, and another regressing the mediator on the exposure and confounders using the Stata *Med4way* command to estimate mediated effects.[29] For objective 4, we will assess the presence of confounding by comparing estimates with and without adjustment. We will use Read codes, which have been previously developed by our team, to identify outcome and covariate records.

Table 2. Confounders

**Confounders and covariates**		**Measures**
Health Behaviours	Smoking status	Categorical (0=never; 1=ex-smoker; 2=current smoker [light: 1-9 cigarettes/day; moderate: 10-19 cigarettes/day; heavy: 20+ cigarettes/day]). This is coded in GP records. We will use the most recent recorded value of smoking status at the study start date.
	Alcohol use	Continuous (units per week). This is coded in GP records. We will use the most recent recorded value of alcohol use at the study start date.

For peer review only - http://bmjopen.bmj.com/site/about/guidelines.xhtml

Demographics	Socioeconomic status (SES)[a]	Categorical (quintiles)
	Ethnicity[b]	Categorical (White; Asian or Asian British; Black or Black British; other ethnic group, mixed)
	Sex[b]	Binary (0=male; 1=female)
	Age at study entry, in years	Continuous
Physical and/or medical factors	Baseline weight status (body mass index (BMI))	Categorical (normal weight [BMI 18.5-24.9 kg/m^2]; overweight [25.0-29.9]; obese [30.0-34.9]; severe obesity [35.0-35.9]; morbid obesity [40.0-44.9]
	Psychotropic medication	Categorical (antipsychotics [first- and second-generation]; antidepressants; mood stabilisers]
	Cardio-metabolic medication	Categorical (blood glucose lowering medications; antihypertensive treatments, statins)
We include the following additional variables for objectives 3 and 4 only		
CVD risk factors	Total cholesterol/HDL ratio	Continuous
	Systolic and diastolic blood pressure	Continuous
	Glycaemia	HbA1c/fasting blood glucose (FBG)

[a]This variable is grouped according to practice level Index of Multiple Deprivation (IMD). This is an indicator of material deprivation based on income; employment; health deprivation and disability; education, skills, and training; crime; barriers to housing and services; and living environment. It is categorised as quintiles 1-5 with 1 being the most deprived and 5 being the least deprived.

[b]This variable is grouped according to recommendations by the UK Office for National Statistics.[30,31]

i. Variable Library

Exposures, outcomes, and covariates of interest will be identified using medical coding (Read/SNOMED/EMIS), product coding (DM+D), and recording of patient death or transfer out of a participating practice in CPRD Aurum. The final definitions and code library has been drawn up and validated through consensus agreement amongst all investigators.

j. Sample Size Calculation

The estimated median follow-up time for the *exposed* with a weight Read code in their record in the one-year *before* the index date is 3 · 2 years (mean: 4.6), which is based on our feasibility work for this project. We assumed a 1% annual incidence rate of CVD meaning 3% of the study population would develop CVD over the 3 · 2 years of follow-up.[32] We also assumed a relative risk (RR) of 1 · 3 for CVD in the *exposed* versus *unexposed*.[33] The *n* ratio was set at 0 · 25, which achieved the best balance between statistical power and data cost. Our calculation suggested a minimum sample size of *n*=3,907 at 80% power to detect an RR of 1 · 3, alpha $\leq$0.05%. Our feasibility estimates indicate that there are 135,870 patients with SMI in CPRD Aurum, meaning the study is sufficiently powered to detect small effects.

For peer review only - http://bmjopen.bmj.com/site/about/guidelines.xhtml

k. Statistical analysis

Analyses will be conducted in Stata using an estimation approach. We do not intend to impute missing data. The entry date into the analysis is the index date. The outcome date is the earliest of: (i) the date of death, or (ii) date of diagnosis of the outcome of interest. For objectives 1 and 2, we will use all repeated measures and run separate models for each analyses. For objective 3, we will use the first recorded diagnosis of the outcome of interest rather than recurrent events. Patients who do not have the outcome of interest will be censored at the earliest of: (i) date of death, (ii) date of leaving the practice, (iii) date of the latest download of data, or (iv) the study end date.

Descriptive summaries:

Frequency matching will reduce potential confounding and balance the baseline characteristics of the *exposed* and *unexposed* on age, sex, practice, and calendar year. Adequacy of matching will be confirmed by comparing means using independent *t*-tests. Descriptive statistics will describe the baseline characteristics of patients in the cohort. Categorical data will be presented as frequency and percentages, and continuous variables will be summarised using descriptive statistics (mean, standard deviation [SD], or median and interquartile range). Comparisons between the groups will be made using χ^2 tests and *t*-tests (or Wilcoxon rank-sum tests) as appropriate. Length of exposure and the most commonly prescribed class of psychotropic for each patient will be summarised. The causes of death will be categorised as CVD and all-cause mortality.

Primary analyses:

For objectives 1 and 2, the outcome will be visually inspected by plotting trajectories of weight and cardio-metabolic risk factor change using all repeated measurements for patients with SMI versus patients without SMI. For these analyses, we will use a mixed-effects linear regression model including terms for SMI, time, and their interaction to estimate regression coefficients with 95% CIs by SMI status. Models incorporating psychotropic medications will analogously include medication-time multiplicative interaction terms. We will include a random effect for individual and practice identifier to account for intra-class correlation (ICC) resulting from common practice membership. Diagnostic tests will be conducted to confirm linear regression assumptions are met including linearity of residuals, residual normality using quantile-quantile (Q-Q) plots, and residual heteroscedasticity using residual versus fitted value plots. We will assess for exploratory sub-group effects by adding interactions (e.g., by age, sex, ethnicity, and SES) and, if significant, we will assess the strength of the associations in separate sub-groups for clarity. For objective 2, we will model the association between weight and cardio-metabolic change using restricted cubic splines to allow for non-linear associations. For objective 3, we will use Kaplan-Meier survival estimates for the exposed and unexposed groups to estimate the distribution of time from index date to total and fatal CVD and mortality (all-cause). The statistical equivalence of the two curves will be examined using the log-rank test. This analysis will use a survival from index date between the exposed and unexposed groups using an adjusted Cox proportional hazards regression model with follow-up time as the timescale. Restricted cubic splines will be computed with five knots to visually explore non-linear associations between the exposure and the outcomes. For objective 4, we will use a Poisson regression examining person-time to estimate the rate at which patients who received weight management services over the study period in patients with SMI compared with patients who do not have SMI, adjusting for confounders and BMI.

For peer review only - http://bmjopen.bmj.com/site/about/guidelines.xhtml

Sensitivity and secondary analyses:

If necessary, data on missing confounders and covariates will be addressed by adjusting for missingness as a category. Sensitivity analyses will examine the impact of running the analyses with everyone included as discussed above and confined only to those with complete data. A second sensitivity analysis will be performed excluding patients that experience the outcome within two years of the SMI Read code to avoid reversed causality from other underlying disease.

Ethics and dissemination

This study has been approved by the Independent Scientific Advisory Committee (ISAC) to the Medicines and Healthcare Products Regulatory Agency (reference: 20_000186). To guarantee the confidentiality of patient information, only the authors will have access to the data during the study. The full statistical code will be available from the authors after the publication of the results. The results of this study will be presented in scientific conferences and published in peer-reviewed journals.

Discussion

In the UK, the NHS Long Term Plan commits the health service to greater action on reducing inequalities for the most overlooked groups. This study responds to this national agenda by focusing on the major cause of death for people with SMI – cardiovascular disease. We plan to leverage the power of a large UK primary care database to provide new insight on how these actions may be achieved.

Our results will estimate weight change, its risk factors including psychotropic medication, and its management in primary care as a preventative target for CVD. This could inform medication choice for people with SMI and clinicians, particularly if there are meaningful differences between drugs. In addition, our results will estimate the change in cardio-metabolic factors in people with SMI relative to those without SMI. This will be valuable to policy makers because these are key risk factors for CVD that are being addressed through the NHS Long Term Plan. Furthermore, identifying the factors underlying the higher incidence of CVD will help us understand the degree to which these factors are valuable therapeutic targets. Lastly, understanding whether people with SMI are being offered support to manage their weight will inform national initiatives to overcome therapeutic nihilism. It is also important to understand whether there are differences in response between people with SMI and the general population, which would inform us about whether interventions need to be tailored to people with SMI for more personalised care. Ultimately, the results will offer a foundation to assess the degree to which the current focus on CVD prevention in people with SMI is having clinical benefits.

Since this study is observational, there are inherent strengths and limitations that are discussed. A strength of the study is its use of a representative, validated, and sizeable dataset. Furthermore, the CPRD Aurum is an appropriate data source utilising primary care records. This will minimise selection, recall, and respondent bias since UK general practices tend to have good levels of accuracy in recording prescriptions and clinical diagnoses. The results will also have direct and important applications to international guidelines on patient healthcare management, as well as to British primary care physicians, mental health services, and members of the public with experience of SMI to

For peer review only

initiate individual action. Lastly, the longitudinal design is useful in addressing important clinical questions not usually possible within RCTs, and a matched-cohort improves efficiency since we will not need to include all controls, whilst tackling confounding.

Since the study is descriptive, any association observed would not imply an SMI diagnosis is causative of adverse physical health outcomes. However, we might assume the greater the magnitude of the observed association the less likely it is due to bias and more likely due to a causal process.[34] Furthermore, diet and physical activity are unmeasured confounders because they are unobserved in CPRD Aurum. Similarly, other unmeasured confounders like poverty may place people with SMI at increased risk of homelessness, which has consequences on healthcare access and provision. We address this potential confounding by linking to patient level IMD as a proxy measure of SES, but acknowledge this may not fully capture deprivation and leaves room for residual confounding. Moreover, information bias may be present since the CPRD Aurum relies on a GP recording of health outcomes. Furthermore, we can only examine primary care prescriptions. Any prescribing solely within secondary care may not be included as this was not historically routinely recorded in GP records. Thus, it is possible we will miss the first prescription(s). Lastly, physical health checks are not always performed on random samples of the population, and it is possible that more regular testing is done on people with overweight and obesity. We expect that some patients will have incomplete information recorded. However, by determining what proportion of patients do not have appropriate information recorded, we can evaluate the scale of these problems, and highlight the need for improved recording for continuity of care between healthcare professionals.

Acknowledgements

We thank the patient and public Involvement volunteers for their time and for sharing their knowledge. We also thank the patients and practices who contribute to the CPRD database. We acknowledge that HES data that will be used in this analysis will be re-used with permission from NHS Digital, who retain the copyright.

Footnotes

Contributorship statement

CL, FW, PA, and CP conceived the study idea. CL, FW, MS, MG, CB, PA, and CP contributed to the development of the study design and protocol. CL drafted the manuscript. All authors approved the final submitted version.

Funding

This research received no specific grant from any funding agency in the public, commercial or not-for-profit sectors. CL is funded by the Engineering and Physical Sciences Research Council (EPSRC; EP/R513295/1) and the National Institute for Health Research (NIHR) Oxford Biomedical Research Centre (BRC; IS-BRC-1215-20008). MS is supported by the NIHR Oxford BRC. CB is supported by the NIHR Oxford BRC. PA is funded by the NIHR BRC, the NIHR ARC (200172), and is an NIHR senior investigator (NF-SI-0617-10064). CPS is funded through the NIHR ARC. FW is funded by a Wellcome Trust Clinical Doctoral Fellowship (102176/B/13/Z).

For peer review only - http://bmjopen.bmj.com/site/about/guidelines.xhtml

Disclaimer

The views expressed are those of the author(s) and not necessarily those of the NIHR or the Department of Health and Social Care.

Competing interests

PA has conducted a trial funded by Cambridge Weight Plan and has spoken at a seminar at the Royal College of General Practitioners (RCGP) conference that was sponsored by Novo Nordisk. None of these activates led to personal payments to him. All other authors declare no conflict of interests.

Patient consent for publication

Not required.

Data sharing statement

The data was made available by the CPRD obtained under licence from the UK MRHA. Data is available to researchers who are approved by the Independent Scientific Advisory Committee.

References

1. England N. *Improving Physical Healthcare for People Living with Severe Mental Illness (SMI) in Primary Care Guidance for CCGs.*; 2018. https://www.england.nhs.uk/wp-content/uploads/2018/02/improving-physical-health-care-for-smi-in-primary-care.pdf. Accessed May 4, 2021.
2. Hennekens CH, Hennekens AR, Hollar D, Casey DE. Schizophrenia and increased risks of cardiovascular disease. *Am Heart J*. 2005;150(6):1115-1121. doi:10.1016/j.ahj.2005.02.007
3. Osborn DPJ, Levy G, Nazareth I, Petersen I, Islam A, King MB. Relative risk of cardiovascular and cancer mortality in people with severe mental illness from the United Kingdom's General Practice Research Database. *Arch Gen Psychiatry*. 2007;64(2):242-249. doi:10.1001/archpsyc.64.2.242
4. Correll CU, Solmi M, Veronese N, et al. Prevalence, incidence and mortality from cardiovascular disease in patients with pooled and specific severe mental illness: a large-scale meta-analysis of 3,211,768 patients and 113,383,368 controls. *World Psychiatry*. 2017;16(2):163-180. doi:10.1002/wps.20420
5. Yusuf S, Reddy S, Ôunpuu S, Anand S. Global Burden of Cardiovascular Diseases. *Circulation*. 2001;104(22):2746-2753. doi:10.1161/hc4601.099487
6. Saha S, Chant D, McGrath J. A systematic review of mortality in schizophrenia: Is the differential mortality gap worsening over time? *Arch Gen Psychiatry*. 2007;64(10):1123-1131. doi:10.1001/archpsyc.64.10.1123
7. NHS Digital. Health Survey for England 2019 [NS] - NHS Digital. https://digital.nhs.uk/data-and-information/publications/statistical/health-survey-for-england/2019#. Accessed September 30, 2021.
8. National Mental Health Intelligence Network. Severe mental illness (SMI) and physical health inequalities: briefing - GOV.UK. Public Health England. https://www.gov.uk/government/publications/severe-mental-illness-smi-physical-health-inequalities/severe-mental-illness-and-physical-health-inequalities-briefing#fn:1. Published 2018. Accessed November 14, 2019.
9. Leslie WS, Hankey CR, Lean MEJ. Review Weight gain as an adverse effect of some commonly prescribed drugs: a systematic review. *QJM*. 2007;100:395–404. doi:10.1093/qjmed/hcm044
10. Tiihonen J, Lönnqvist J, Wahlbeck K, et al. 11-year follow-up of mortality in patients with schizophrenia: a population-based cohort study (FIN11 study). *Lancet*. 2009;374(9690):620-627. doi:10.1016/S0140-6736(09)60742-X

For peer review only

For peer review only - http://bmjopen.bmj.com/site/about/guidelines.xhtml

11. De Hert M, Correll CU, Cohen D. Do antipsychotic medications reduce or increase mortality in schizophrenia? A critical appraisal of the FIN-11 study. *Schizophr Res*. 2010;117(1):68-74. doi:10.1016/j.schres.2009.12.029
12. Teasdale SB, Ward PB, Samaras K, et al. Dietary intake of people with severe mental illness: Systematic review and meta-analysis. *Br J Psychiatry*. 2019;214(5):251-259. doi:10.1192/bjp.2019.20
13. Psychiatrists RC of PRC of. *Smoking and Mental Health*. London, UK; 2013.
14. Williams J, Ziedonis D, Abanyie F, Steinberg M, Foulds J, Benowitz N. Increased nicotine and cotinine levels in smokers with schizophrenia and schizoaffective disorder is not a metabolic effect. *Schizophr Res*. 2005;79(2-3):323-335. doi:10.1016/J.SCHRES.2005.04.016
15. Kendall KM, John A, Lee SC, et al. Impact of schizophrenia genetic liability on the association between schizophrenia and physical illness: data-linkage study. *BJPsych Open*. 2020;6(6). doi:10.1192/bjo.2020.42
16. Agency for Healthcare Research and Quality. U.S. Preventive Services Task Force. Rockville, MD. https://www.ahrq.gov/cpi/about/otherwebsites/uspstf/index.html. Published 2019.
17. The National Institute for Health and Care Excellence (NICE). Obesity prevention clinical guideline [CG43]. United Kingdom. https://www.nice.org.uk/guidance/CG43. Published 2015.
18. Jolly K, Lewis A, Beach J, et al. Comparison of range of commercial or primary care led weight reduction programmes with minimal intervention control for weight loss in obesity: Lighten Up randomised controlled trial. *BMJ*. 2011;343(7832):1035. doi:10.1136/bmj.d6500
19. Booth HP, Prevost AT, Gulliford MC. Access to weight reduction interventions for overweight and obese patients in UK primary care: Population-based cohort study. *BMJ Open*. 2015;5(1):e006642. doi:10.1136/bmjopen-2014-006642
20. Humphreys K, Blodgett JC, Roberts LW. The exclusion of people with psychiatric disorders from medical research. *J Psychiatr Res*. 2015;70:28-32. doi:10.1016/j.jpsychires.2015.08.005
21. Lieberman JA, Stroup TS, McEvoy JP, et al. Effectiveness of Antipsychotic Drugs in Patients with Chronic Schizophrenia. *N Engl J Med*. 2005;353(12):1209-1223. doi:10.1056/nejmoa051688
22. Jones PB, Barnes TRE, Davies L, et al. Randomized controlled trial of the effect on quality of life of second- vs first-generation antipsychotic drugs in schizophrenia: Cost Utility of the Latest Antipsychotic Drugs in Schizophrenia Study (CUtLASS 1). *Arch Gen Psychiatry*. 2006;63(10):1079-1087. doi:10.1001/archpsyc.63.10.1079
23. Álvarez-Jiménez M, González-Blanch C, Crespo-Facorro B, et al. Antipsychotic-induced weight gain in chronic and first-episode psychotic disorders: A systematic critical reappraisal. *CNS Drugs*. 2008;22(7):547-562. doi:10.2165/00023210-200822070-00002
24. Benchimol E, Smeeth L, Guttmann A, et al. The REporting of studies Conducted using Observational Routinely-collected health Data (RECORD) statement. *PLoS Med*. 2015;12(10). doi:10.1371/JOURNAL.PMED.1001885
25. Hope H, Parisi R, Ashcroft DM, et al. Fertility trends of women with serious mental illness in the United Kingdom 1992–2017: A primary care cohort study using the clinical practice research datalink. *J Affect Disord*. 2020;269:141-147. doi:10.1016/j.jad.2020.03.037
26. Shrier I, Platt RW. Reducing bias through directed acyclic graphs. *BMC Med Res Methodol*. 2008;8:70. doi:10.1186/1471-2288-8-70
27. Merikangas K, He J, Burstein M, et al. Lifetime prevalence of mental disorders in U.S. adolescents: results from the National Comorbidity Survey Replication--Adolescent Supplement (NCS-A). *J Am Acad Child Adolesc Psychiatry*. 2010;49(10):980-989. doi:10.1016/J.JAAC.2010.05.017
28. Schwartz RC. Racial disparities in psychotic disorder diagnosis: A review of empirical literature. *World J Psychiatry*. 2014;4(4):133. doi:10.5498/wjp.v4.i4.133
29. Discacciati A, Bellavia A, Lee JJ, Mazumdar M, Valeri L. Med4way: a Stata command to investigate mediating and interactive mechanisms using the four-way effect decomposition.

For peer review only - http://bmjopen.bmj.com/site/about/guidelines.xhtml

Int J Epidemiol. 2019;48(1):15-20. doi:10.1093/IJE/DYY236
30. Government Statistical Service. Ethnicity harmonised standard. https://gss.civilservice.gov.uk/policy-store/ethnicity/. Published 2019. Accessed August 13, 2021.
31. Government Statistical Service. Sex and gender harmonisation guidance. https://gss.civilservice.gov.uk/policy-store/sex-and-gender/. Published 2019. Accessed August 13, 2021.
32. Fan Z, Wu Y, Shen J, Ji T, Zhan R. Schizophrenia and the risk of cardiovascular diseases: A meta-analysis of thirteen cohort studies. *J Psychiatr Res*. 2013;47(11):1549-1556. doi:10.1016/j.jpsychires.2013.07.011
33. Cunningham R, Poppe K, Peterson D, Every-Palmer S, Soosay I, Jackson R. Prediction of cardiovascular disease risk among people with severe mental illness: A cohort study. *PLoS One*. 2019;14(9). doi:10.1371/journal.pone.0221521
34. Grimes DA, Schulz KF. Bias and causal associations in observational research. *Lancet*. 2002;359(9302):248-252. doi:10.1016/S0140-6736(02)07451-2

For peer review only

For peer review only - http://bmjopen.bmj.com/site/about/guidelines.xhtml

Weight change, cardio-metabolic risk factors, and cardiovascular incidence in people with serious mental illness: protocol of a population-based cohort study in the UK from 1997 – 2020

Supplementary Material

Supplementary material S1. Direct acyclic graphs (DAGs) for the association between SMI and each outcome.

In the following figures, the green box (i.e., SMI status) is the exposure variable. The blue box 'I' is the outcome variable. The grey boxes 'diet' and 'physical activity' are latent variables because they are unobserved in the CPRD Aurum dataset. Causal paths are indicated via green arrows; biasing paths in red; and paths with no influence on the outcome in black.

Figure 1. DAG for the association between SMI and mean weight change (objective 1).

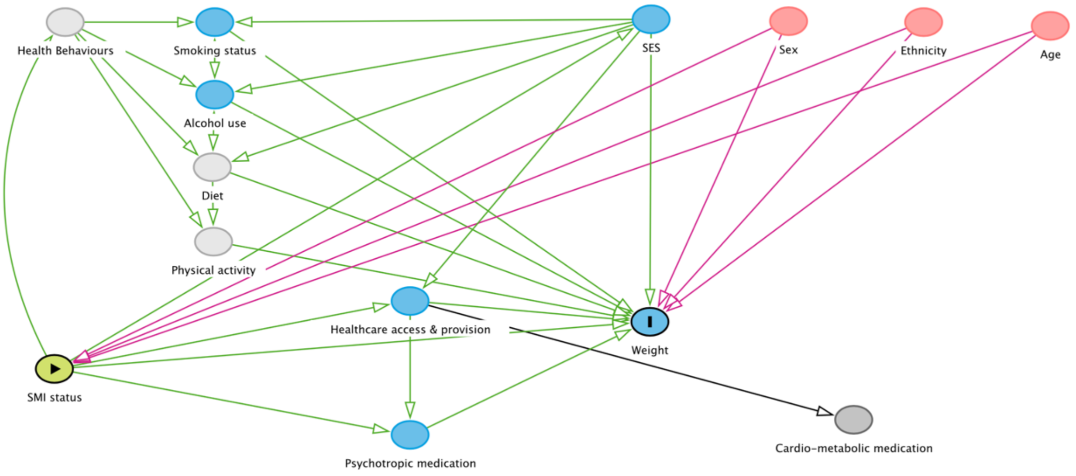

This DAG shows **five** paths between the exposure (i.e., SMI status) and outcome (i.e., weight): the (1) direct path from SMI status to weight; the indirect paths via (2) health behaviours (i.e., smoking status, alcohol use, diet, and physical activity), via (3) socio-economic status (SES), via (4) psychotropic medications, and via (5) healthcare access and provision. All variables on the indirect paths are mediating. There are three confounding paths: the path via age, sex, and ethnicity. Thus, the minimally sufficient adjustment to get the total effect of the exposure on the outcome is to adjust for age, sex, and ethnicity. The direct effect cannot be estimated by covariate adjustment.

Figure 2. DAG for the association between SMI and biomarkers for CVD risk (objective 2).

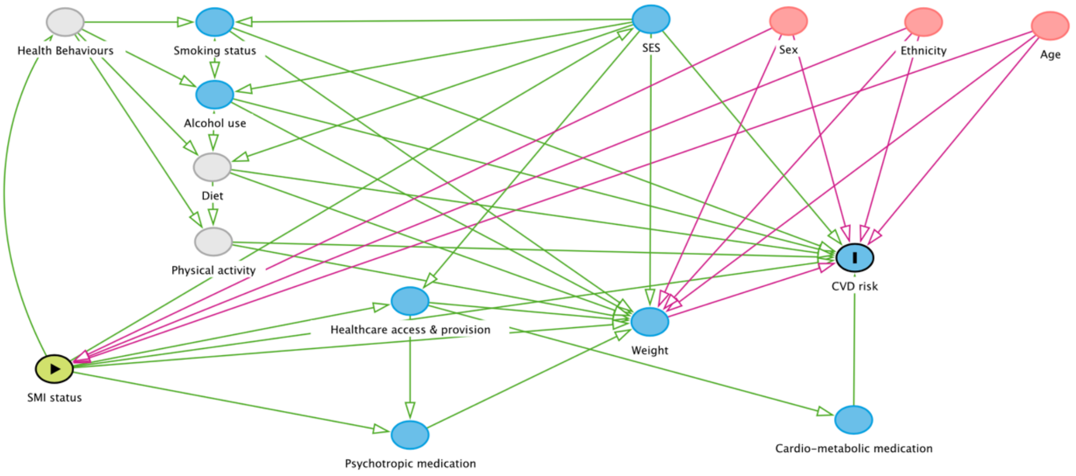

This DAG shows **seven** paths between the exposure (i.e., SMI status) and outcome (i.e., CVD risk): the (1) direct path from SMI status to CVD risk; the indirect paths via (2) health behaviours (i.e., smoking status, alcohol use, diet, physical activity), via (3) SES, via (4) psychotropic medications, via (5) healthcare access and provision, via (6) cardio-metabolic medication, and via (7) weight. There are three confounding paths: the path via age, sex, and ethnicity. Thus, the minimally sufficient adjustment to get the total effect of the exposure on the outcome is to adjust for age, sex, and ethnicity. The direct effect cannot be estimated by covariate adjustment.

Figure 3. DAG for the association between SMI and CVD incidence (objective 3).

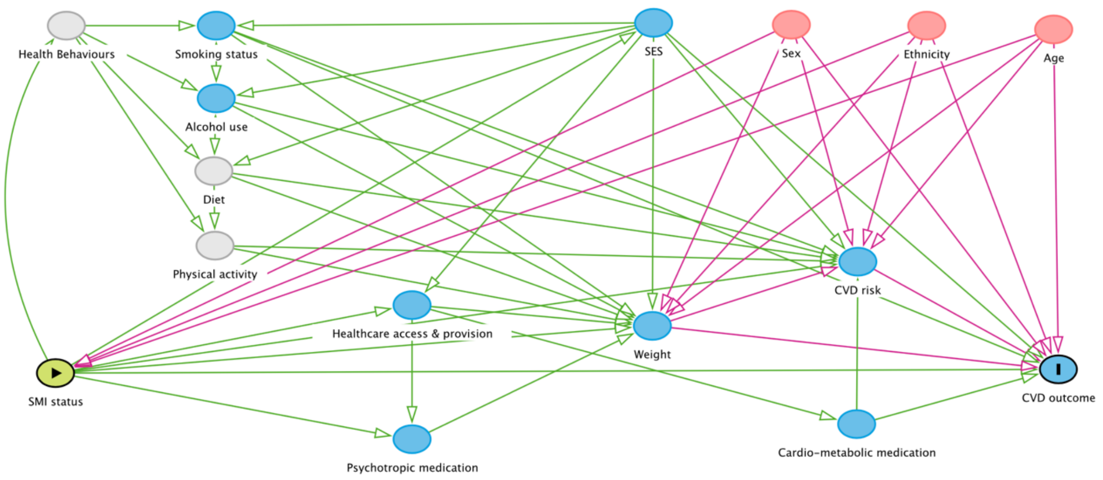

This DAG shows **eight** paths between the exposure (i.e., SMI status) and outcome (i.e., CVD risk): the (1) direct path from SMI status to CVD risk; the indirect paths via (2) health behaviours (i.e., smoking status, alcohol use, diet, and physical activity), via (3) SES, via (4) psychotropic medications, via (5) healthcare access and provision, via (6) cardio-metabolic medication, via (7) weight, and via (8) CVD risk. The minimally sufficient adjustment to get the total effect of the exposure on the outcome is to adjust for age, sex, and ethnicity. The minimally sufficient adjustment for estimating the direct effect of the exposure on the outcome is to adjust for: age, sex, ethnicity, SES, smoking, weight, cardio-metabolic medication, and CVD risk.

For peer review only - http://bmjopen.bmj.com/site/about/guidelines.xhtml

Figure 4. DAG to compare the prevalence of offer and actual referral to weight management services in people with both SMI and obesity with controls (objective 4).

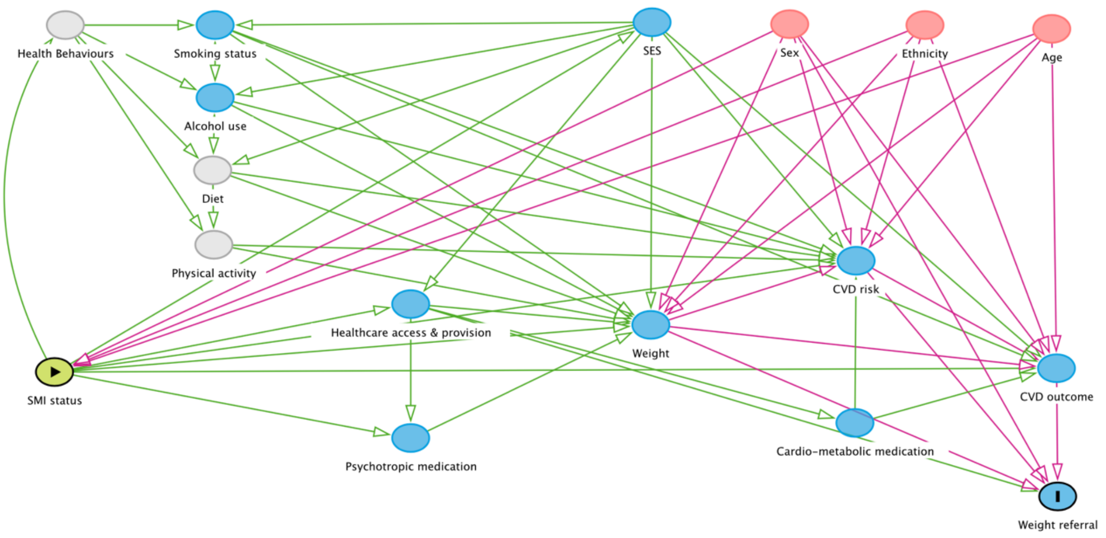

This model does not change the adjustment set.

For peer review only

The RECORD statement – checklist of items, extended from the STROBE statement, that should be reported in observational studies using routinely collected health data.

	Item No.	STROBE items	Location in manuscript where items are reported	RECORD items	Location in manuscript where items are reported
**Title and abstract**					
	1	(a) Indicate the study's design with a commonly used term in the title or the abstract (b) Provide in the abstract an informative and balanced summary of what was done and what was found	(a): Page 1 – Title; Page 2 – Abstract<br><br>(b): Not applicable because this manuscript reports a protocol	RECORD 1.1: The type of data used should be specified in the title or abstract. When possible, the name of the databases used should be included.<br><br>RECORD 1.2: If applicable, the geographic region and timeframe within which the study took place should be reported in the title or abstract.<br><br>RECORD 1.3: If linkage between databases was conducted for the study, this should be clearly stated in the title or abstract.	(1.1): Page 2 - Abstract<br><br>(1.2): Page 2 - Abstract<br><br>(1.3): Page 2 - Abstract
**Introduction**					
Background rationale	2	Explain the scientific background and rationale for the investigation being reported	Page 3 – Background		
Objectives	3	State specific objectives, including any prespecified hypotheses	Page 4 – Background		
**Methods**					
Study Design	4	Present key elements of study design early in the paper	Methods and analysis:<br>• Page 4 - Design		
Setting	5	Describe the setting, locations, and relevant dates, including periods of recruitment, exposure, follow-up, and data collection	Methods and analysis:<br>• Settings, locations, data collection: Page 4 - Data Source<br>• Periods of recruitment: Page 5 - Study Cohort<br>• Exposure: Page 6 - Exposures<br>• Follow-up: Page 5 –		

For peer review only

			Duration of Follow-Up		
Participants	6	*(a) Cohort study* - Give the eligibility criteria, and the sources and methods of selection of participants. Describe methods of follow-up<br>*Case-control study* - Give the eligibility criteria, and the sources and methods of case ascertainment and control selection. Give the rationale for the choice of cases and controls<br>*Cross-sectional study* - Give the eligibility criteria, and the sources and methods of selection of participants<br><br>*(b) Cohort study* - For matched studies, give matching criteria and number of exposed and unexposed<br>*Case-control study* - For matched studies, give matching criteria and the number of controls per case	(a): Page 5 – Study Cohort<br><br>(b): Page 5 – Comparison Group	RECORD 6.1: The methods of study population selection (such as codes or algorithms used to identify subjects) should be listed in detail. If this is not possible, an explanation should be provided.<br><br>RECORD 6.2: Any validation studies of the codes or algorithms used to select the population should be referenced. If validation was conducted for this study and not published elsewhere, detailed methods and results should be provided.<br><br>RECORD 6.3: If the study involved linkage of databases, consider use of a flow diagram or other graphical display to demonstrate the data linkage process, including the number of individuals with linked data at each stage.	(6.1): Page 5 – Study Cohort<br><br>(6.2): Not done because this manuscript reports a protocol. We will do this for any output papers.<br><br>(6.3): Considered but not thought necessary.
Variables	7	Clearly define all outcomes, exposures, predictors, potential confounders, and effect modifiers. Give diagnostic criteria, if applicable.	Methods and analysis:<br>• Exposures: Page 6 – Exposures<br>• Potential confounders and effect modifiers – Page 7 – Confounding	RECORD 7.1: A complete list of codes and algorithms used to classify exposures, outcomes, confounders, and effect modifiers should be provided. If these cannot be reported, an explanation should be provided.	(7.1): Not provided because this manuscript reports a protocol. We will provide this for any output papers.
Data sources/ measurement	8	For each variable of interest, give sources of data and details of methods of assessment (measurement). Describe comparability of assessment methods if there is more than one group	Not considered		
Bias	9	Describe any efforts to address potential sources of bias	Page 7 - Confounding		
Study size	10	Explain how the study size was arrived at	Page 8 – Sample size calculation		
Quantitative variables	11	Explain how quantitative variables were handled in the analyses. If	Page 9 – Primary analyses		

For peer review only

		applicable, describe which groupings were chosen, and why			
Statistical methods	12	(a) Describe all statistical methods, including those used to control for confounding<br>(b) Describe any methods used to examine subgroups and interactions<br>(c) Explain how missing data were addressed<br>(d) *Cohort study* - If applicable, explain how loss to follow-up was addressed<br>*Case-control study* - If applicable, explain how matching of cases and controls was addressed<br>*Cross-sectional study* - If applicable, describe analytical methods taking account of sampling strategy<br>(e) Describe any sensitivity analyses	(a): Page 9 – Statistical methods (primary analyses)<br><br>(b): Page 9 – Statistical methods (primary analyses)<br><br>(b): Page 9 – Statistical methods (sensitivity and secondary analyses)		
Data access and cleaning methods		..		RECORD 12.1: Authors should describe the extent to which the investigators had access to the database population used to create the study population.<br><br>RECORD 12.2: Authors should provide information on the data cleaning methods used in the study.	(12.1): Not considered in the manuscript. But bull access will available.<br><br>(12.2): Not yet performed.
Linkage		..		RECORD 12.3: State whether the study included person-level, institutional-level, or other data linkage across two or more databases. The methods of linkage and methods of linkage quality evaluation should be provided.	(12.3): Page 5 – Data Linkage
**Results**					
Participants	13	(a) Report the numbers of individuals at each stage of the study (*e.g.*, numbers potentially eligible, examined for eligibility, confirmed eligible, included in the study, completing follow-up, and analysed)<br>(b) Give reasons for non-participation at each stage.<br>(c) Consider use of a flow diagram	(a-c): Not applicable	RECORD 13.1: Describe in detail the selection of the persons included in the study (*i.e.*, study population selection) including filtering based on data quality, data availability and linkage. The selection of included persons can be described in the text and/or by means of the study flow diagram.	(13.1): Not applicable
Descriptive data	14	(a) Give characteristics of study participants (*e.g.*, demographic, clinical, social) and information on exposures and potential confounders	(a-c): Not applicable		

For peer review only

		(b) Indicate the number of participants with missing data for each variable of interest<br>(c) *Cohort study* - summarise follow-up time (*e.g.*, average and total amount)			
Outcome data	15	*Cohort study* - Report numbers of outcome events or summary measures over time<br>*Case-control study* - Report numbers in each exposure category, or summary measures of exposure<br>*Cross-sectional study* - Report numbers of outcome events or summary measures	Not applicable		
Main results	16	(a) Give unadjusted estimates and, if applicable, confounder-adjusted estimates and their precision (e.g., 95% confidence interval). Make clear which confounders were adjusted for and why they were included<br>(b) Report category boundaries when continuous variables were categorized<br>(c) If relevant, consider translating estimates of relative risk into absolute risk for a meaningful time period	(a-c): Not applicable		
Other analyses	17	Report other analyses done—e.g., analyses of subgroups and interactions, and sensitivity analyses	Not applicable		
Key results	18	Summarise key results with reference to study objectives	Not applicable		
Limitations	19	Discuss limitations of the study, taking into account sources of potential bias or imprecision. Discuss both direction and magnitude of any potential bias	Page 9-10 - Discussion	RECORD 19.1: Discuss the implications of using data that were not created or collected to answer the specific research question(s). Include discussion of misclassification bias, unmeasured confounding, missing data, and changing eligibility over time, as they pertain to the study being reported.	Not applicable
Interpretation	20	Give a cautious overall interpretation of results considering objectives, limitations, multiplicity of analyses, results from similar studies, and other relevant evidence	Not applicable		
Generalisability	21	Discuss the generalisability (external validity) of the study results	Page 9-10 - Discussion		
**Other Information**					

Funding	22	Give the source of funding and the role of the funders for the present study and, if applicable, for the original study on which the present article is based	Page 10 – Acknowledgements (Funding)		
Accessibility of protocol, raw data, and programming code		..		RECORD 22.1: Authors should provide information on how to access any supplemental information such as the study protocol, raw data, or programming code.	Page 10 – Acknowledgements (Ethics and dissemination)

*Reference: Benchimol EI, Smeeth L, Guttmann A, Harron K, Moher D, Petersen I, Sørensen HT, von Elm E, Langan SM, the RECORD Working Committee. The REporting of studies Conducted using Observational Routinely-collected health Data (RECORD) Statement. *PLoS Medicine* 2015; in press.

*Checklist is protected under Creative Commons Attribution (CC BY) license.

For peer review only
